# Supplementary material for: Pathways to reduced physical intimate partner violence among women in north-western Tanzania: Evidence from two cluster randomised trials of the MAISHA intervention
Source: PLOS Glob Public Health. 2023 Nov 13;3(11):e0002497. doi: 10.1371/journal.pgph.0002497 (PMC10642778; doi:10.1371/journal.pgph.0002497)
Supplement: S1 Fig — (PDF) [file pgph.0002497.s002.pdf]

Context

Community
Urban environment

Normative attitudes to gender roles

Normative attitudes toward IPV

Acceptability of Violence

Intimate Relationships
Relationship power dynamics

Patterns of communication

History of abuse, prior levels of violence

Extra-spousal sex partners

Individual
Attitudes and acceptability of violence and other abuses

Microfinance

Financial uncertainty and insecurity

Economic Opportunities

Time pressures

Intervention

Group-based microfinance loans

Participatory gender training encompassing:
Understanding gender;

Act like a man, act like a woman;

Healthy and unhealthy relationships;

Power and control in relationships;

Negotiating men's and women's roles inside and outside the home;

Communicating assertively with your partner;

Violence;

Setting personal boundaries;

Non-violent ways to reduce conflict;

Empowering change.

Initial Outcomes

Knowledge
Identify inequitable and harmful gender norms that exist in the community

Recognise and understand different forms of violence

Recognise and understand why controlling behavior and other abuses are unhealthy in a romantic relationship

Awareness
Normative attitudes to gender roles and their consequences in communities

Normative attitudes to IPV and their impact on individuals, families and communities

Group dynamics
Increased group stability

Increased group support and social capital

Improved communication

Increased status in community

Decreased stigma – poverty, violence and education

Critical thinking and personal reflection
Attitude to gender roles – redefining inequitable and harmful gender norms

Attitude to controlling behaviour and other abuses

Healthy and unhealthy romantic relationships

Healthy and unhealthy expressions of power

Intermediate Outcomes

Skills
Identify, set and manage personal boundaries

Negotiate division of labour inside and outside the home

Communicate using an assertive communication style

Intimate relationships
Increased communication, joint decision-making with partner and trust

Give clear sexual consent

Prevent, negotiate, and resolve conflict using non-violent means

Individual
Increased confidence, self- efficacy and authority

Ability to resist and challenge social pressure to conform to inequitable gender norms that support violent behaviour

Economic
Increased financial autonomy and status in household

Reduced household economic stress

Longer-term Outcomes

Group capacity
Supportive environment

Higher proportion of women experiencing violence disclosing it to others

Ability to identify when and how women can obtain support against violence (e.g. health, social, legal)

Intimate relationships
Greater equality in relationship

Mutual respect

Improved communication

Reduced risk behaviours

Individual
Enhanced ability to prevent and respond to IPV

Improvement in physical and mental health and general well-being

Impact

Reduced levels of physical and/or sexual IPV and other forms of abuse experienced by women

Improved gender equality attitudes and experiences
