# Supplementary material for: Pathways to reduced physical intimate partner violence among women in north-western Tanzania: Evidence from two cluster randomised trials of the MAISHA intervention
Source: PLOS Glob Public Health. 2023 Nov 13;3(11):e0002497. doi: 10.1371/journal.pgph.0002497 (PMC10642778; doi:10.1371/journal.pgph.0002497)
Supplement: S1 Table — (DOCX) [file pgph.0002497.s003.docx]

S1 Table: Questions used to construct potential mediator variables

| **Variable** | **How measured** |
| --- | --- |
| ***Individual-level attitudes (among all ever-partnered women)*** | |
| Attitudes accepting of IPV  (Yes; No) | CODED AS ‘YES’ IF:  Reports that she ‘strongly agrees’ or ‘agrees’ that a man has good reason to hit his wife in at least one of the following scenarios:   - She does not complete her household work to his satisfaction - She disobeys him - She refuses to have sexual intercourse with him - She protests because he has other girlfriends - He suspects that she is unfaithful in marriage   He finds out that she has been unfaithful in marriage  CODED AS ‘NO’ IF ‘STRONGLY DISAGREES’ OR ‘DISAGREES’ WITH ALL |
| Believes a woman is obliged to have sex with her husband  (Yes; No) | CODED AS ‘YES’ IF:  Reports that she ‘strongly agrees’ or ‘agrees’ that:   - It’s a wife’s obligation to have sex with her husband even if she doesn’t want to   CODED AS ‘NO’ IF ‘STRONGLY DISAGREES’ OR ‘DISAGREES’ WITH STATEMENT |
| Believes a woman should obey her husband  (Yes; No) | CODED AS ‘YES’ IF:  Reports that she ‘strongly agrees’ or ‘agrees’ that:   - A woman should obey her husband’s wishes even if she disagrees   CODED AS ‘NO’ IF ‘STRONGLY DISAGREES’ OR ‘DISAGREES’ WITH STATEMENT |
| Believes the man must be the primary provider for the family  (Yes; No) | CODED AS ‘YES’ IF:  Reports that she ‘strongly agrees’ or ‘agrees’ that:   - It **must** be the man who is the primary provider for the family.   CODED AS ‘NO’ IF ‘STRONGLY DISAGREES’ OR ‘DISAGREES’ WITH STATEMENT |
| ***Social participation/ help-seeking/ bystander action (among all ever-partnered women)*** | |
| Participation in community meetings  (Yes; No) | CODED AS ‘YES’ IF:  Responds ‘yes’ to question:  In the past 2 years, have you participated in a meeting, march, rally or gathering aiming to raise awareness and mobilize people around an issue that is important in your community? For example, HIV, rights for albinos or women, etc.  CODED AS ‘NO’ IF RESPONDS ‘NO’ TO QUESTION |
| Very comfortable seeking help for IPV (hypothetical)  (Yes; No) | CODED AS ‘YES’ IF:  Responds ‘very comfortable’ to question:   - If you were abused by your husband or partner, how comfortable would you feel seeking support from a trusted friend or neighbor? Would you say: Very comfortable; Comfortable but would need encouragement; Not comfortable at all; Don’t know?   CODED AS ‘NO’ IF RESPONDS ‘COMFORTABLE BUT WOULD NEED ENCOURAGEMENT’, ‘NOT COMFORTABLE AT ALL’, OR ‘DON’T KNOW’ |
| Group-level confidence to intervene in cases of IPV (hypothetical)  (Continuous variable) | Percentage of the respondent’s group (microfinance group or newly formed group) that report that they would be ‘very confident’ in response to the following question:   - How confident are you in your ability to intervene in cases of domestic violence? Would you say: Very confident; Confident but would need to be encouraged; Not confident at all; Don’t know? |
| ***Relationship dynamics (among women partnered in past year)*** | |
| Confident to assert an opinion different to partner’s  (Yes; No) | CODED AS ‘YES’ IF:  Responds ‘very confident’ to:   - How confident do you feel to assert your own opinion if it is different from that of your husband? (Very confident; Confident but would need to be encouraged; Not confident at all; Don’t know)   CODED AS ‘NO’ IF RESPONDS ‘CONFIDENT BUT WOULD NEED TO BE ENCOURAGED’, ‘NOT CONFIDENT AT ALL’, OR ‘DON’T KNOW’ |
| Good communication with partner  (Yes; No) | CODED AS ‘YES’ IF:  Answers ‘a few times’ or ‘many times’ to all of the following questions:  During the last 12 months, did you and your partner discuss the following topics together:   - things that happened to you during the day? Would you say never, once, a few times, or many times? - things that happened to him in the day? Would you say never, once, a few times, or many times? - your worries or feelings? Would you say never, once, a few times, or many times? - his worries or feelings? Would you say never, once, a few times, or many times?   CODED AS ‘NO’ IF RESPONDS ‘NEVER’ OR ‘ONCE’ TO ANY OF THE QUESTIONS |
| Partner often suspicious that she is unfaithful (Past year)  (Yes; No) | CODED AS ‘YES’ IF:  Answers ‘yes’ to the following question, in relation to the past 12 months:  Thinking about your (current or most recent or past) husband/partner, would you say it is generally true that he is often suspicious that you are unfaithful?  CODED AS ‘NO’ IF ANSWERS ‘NO’ TO QUESTION |
| Have argued with partner over her not fulfilling her role as wife and mother (past year)  (Yes; No) | CODED AS ‘YES’ IF:  Answers ‘yes’ to question:  In your relationship with your current/most recent partner, would you say that you have quarreled in the past 12 months?  *and*  Answers ‘a few times’ or ‘many times’ to:  How often have you quarreled about accusations that you are not fulfilling your responsibilities as wife and mother? (Never; Once; A few times; Many times)  CODED AS ‘NO’ IF ANSWERS ‘NO’ TO ANY QUARRELING IN PAST 12 MONTHS OR ‘NEVER’/’ONCE’ TO QUARRELING ABOUT THIS TOPIC |
| Have argued with partner over his inability/ unwillingness to provide for the family (past year)  (Yes; No) | CODED AS ‘YES’ IF:  Answers ‘yes’ to question:  In your relationship with your current/most recent partner, would you say that you have quarreled in the past 12 months?  *and*  Answers ‘a few times’ or ‘many times’ to:  How often have you quarreled about his inability or unwillingness to provide for the family  CODED AS ‘NO’ IF ANSWERS ‘NO’ TO ANY QUARRELING IN PAST 12 MONTHS OR ‘NEVER’/’ONCE’ TO QUARRELING ABOUT THIS TOPIC |
| Have argued with partner over her disobeying/disrespecting him (past year)  (Yes; No) | CODED AS ‘YES’ IF:  Answers ‘yes’ to question:  In your relationship with your current/most recent partner, would you say that you have quarreled in the past 12 months?  *and*  Answers ‘a few times’ or ‘many times’ to:  How often have you quarreled about you disobeying your partner or treating him disrespectfully.  CODED AS ‘NO’ IF ANSWERS ‘NO’ TO ANY QUARRELING IN PAST 12 MONTHS OR ‘NEVER’/’ONCE’ TO QUARRELING ABOUT THIS TOPIC |
| Have argued with partner over him treating her or her children disrespectfully (past year)  (Yes; No) | CODED AS ‘YES’ IF:  Answers ‘yes’ to question:  In your relationship with your current/most recent partner, would you say that you have quarreled in the past 12 months?  *and*  Answers ‘a few times’ or ‘many times’ to:  How often have you quarreled about him treating you or your children disrespectfully.  CODED AS ‘NO’ IF ANSWERS ‘NO’ TO ANY QUARRELING IN PAST 12 MONTHS OR ‘NEVER’/’ONCE’ TO QUARRELING ABOUT THIS TOPIC |
| Have argued with partner at all about any topic (past year)  (Yes; No) | CODED AS ‘YES’ IF:  Answers ‘yes’ to question:  In your relationship with your current/most recent partner, would you say that you have quarreled in the past 12 months?  CODED AS ‘NO’ IF ANSWERS ‘NO’ TO ANY QUARRELING IN PAST 12 MONTHS |
| ***Propensity to be in a relationship (among those partnered in past year at baseline)*** | |
| Separated/changed partner since baseline | CODED AS ‘YES’ IF:  Reports having had a partner in the past year at baseline, but not having had a partner in the past year at follow-up  *Or*  Reports having had a partner in the past year at both baseline and follow-up, but responds ‘no’ to the question: Is this the same man as when you were interviewed two years ago?  CODED AS ‘NO’ IF HAD A PARTNER IN PAST YEAR AT BASELINE AND FOLLOW-UP, AND RESPONDS ‘YES’ TO IT BEING THE SAME MAN |
