# Supplementary material for: Pathways to reduced physical intimate partner violence among women in north-western Tanzania: Evidence from two cluster randomised trials of the MAISHA intervention
Source: PLOS Glob Public Health. 2023 Nov 13;3(11):e0002497. doi: 10.1371/journal.pgph.0002497 (PMC10642778; doi:10.1371/journal.pgph.0002497)
Supplement: S2 Table — (DOCX) [file pgph.0002497.s004.docx]

S2 Table: Baseline characteristics of participants in CRT01 and CRT02, disaggregated by trial arm

|  |  | **CRT01** | | | **CRT02** | | |
| --- | --- | --- | --- | --- | --- | --- | --- |
|  |  | **Intervention group** | **Control group** | **χ^2^ p-value** | **Intervention group** | **Control group** | **χ^2^ p-value** |
|  |  | (N=485)  n (%)* | (N=434)  n (%)* |  | (N=550)  n (%) | (N=575)  n (%) |  |
| Age (yrs) | *Mean (sd) [range]* | 39·2 (9.4) [20-70] | 40.1 (9.5) [19-70] | 0.185** | 33.2 (8.1) [20-50] | 33.0 (8.0) [18-50] | 0.648** |
| Marital status | *Married* | 353 (73%) | 322 (74%) |  | 445 (81%) | 461 (80%) |  |
|  | *Divorced/separated* | 69 (14%) | 65 (15%) |  | 52 (9%) | 62 (11%) |  |
|  | *Widowed* | 50 (10%) | 37 (9%) |  | 26 (5%) | 26 (5%) |  |
|  | *Never married* | 13 (3%) | 10 (2%) | 0.789 | 27 (5%) | 26 (5%) | 0.891 |
| Partnered in past year |  | 426 (88%) | 379 (87%) | 0.816 | 503 (91%) | 527 (92%) | 0.905 |
| Highest level of education | *None/incomplete primary* | 81 (17%) | 50 (12%) |  | 100 (18%) | 114 (20%) |  |
|  | *Completed primary* | 325 (67%) | 266 (61%) |  | 344 (63%) | 336 (58%) |  |
|  | *Secondary/higher* | 79 (16%) | 118 (27%) | <0.001 | 106 (19%) | 125 (22%) | 0.365 |
| Number of children (<18yrs) | *None* | 31 (6%) | 28 (6%) |  | 23 (4%) | 29 (5%) |  |
|  | *1-2* | 162 (33%) | 139 (32%) |  | 186 (34%) | 234 (41%) |  |
|  | *3-4* | 189 (39%) | 179 (41%) |  | 225 (41%) | 213 (37%) |  |
|  | *5+* | 103 (21%) | 88 (20%) | 0.912 | 116 (21%) | 99 (17%) | 0.063 |
| Respondent worked for money during past 12 months |  | 470 (97%) | 421 (97%) | 0.932 | 442 (80%) | 461 (80%) | 0.936 |
| Reported physical IPV | *Ever* | 261 (54%) | 226 (52%) | 0.598 | 320 (58%) | 325 (57%) | 0.572 |
|  | *Past year* | 94 (19%) | 78 (18%) | 0.585 | 141 (26%) | 140 (24%) | 0.617 |
| Reported sexual IPV | *Ever* | 190 (39%) | 135 (31%) | 0.011 | 215 (39%) | 239 (42%) | 0.398 |
|  | *Past year* | 96 (20%) | 55 (13%) | 0.004 | 105 (19%) | 130 (23%) | 0.147 |
| Reported emotional abuse | *Ever* | 339 (70%) | 292 (67%) | 0.393 | 407 (74%) | 406 (71%) | 0.202 |
|  | *Past year* | 207 (43%) | 159 (37%) | 0.062 | 249 (45%) | 252 (44%) | 0.624 |

*Except for respondent’s age where mean, standard deviation and range are presented

**From t-test of association
