# Supplementary material for: Pathways to reduced physical intimate partner violence among women in north-western Tanzania: Evidence from two cluster randomised trials of the MAISHA intervention
Source: PLOS Glob Public Health. 2023 Nov 13;3(11):e0002497. doi: 10.1371/journal.pgph.0002497 (PMC10642778; doi:10.1371/journal.pgph.0002497)
Supplement: S3 Table — (DOCX) [file pgph.0002497.s005.docx]

S3 Table: Odds ratios comparing past year experience of IPV in CRT02 versus CRT01, before and after adjustment for respondent’s age

| Past year experience of IPV | Crude OR* (95%CI) | Age-adjusted OR* (95%CI) |
| --- | --- | --- |
| Physical | 1.45 (1.17 – 1.79) | 1.09 (0.87 – 1.37) |
| Sexual | 1.35 (1.07 – 1.69) | 1.08 (0.85 – 1.38) |
| Emotional | 1.22 (1.02 – 1.45) | 0.97 (0.80 – 1.18) |
| Economic | 1.22 (1.01 – 1.47) | 1.21 (0.99 – 1.48) |

*OR compares odds of IPV in component B versus A
