# Supplementary material for: Pathways to reduced physical intimate partner violence among women in north-western Tanzania: Evidence from two cluster randomised trials of the MAISHA intervention
Source: PLOS Glob Public Health. 2023 Nov 13;3(11):e0002497. doi: 10.1371/journal.pgph.0002497 (PMC10642778; doi:10.1371/journal.pgph.0002497)
Supplement: S4 Table — (DOCX) [file pgph.0002497.s006.docx]

S4 Table: Adjusted odds ratios of intervention impact on past year physical IPV among different sub-groups of women in CRT02

|  | Prevalence of past year IPV at follow-up  n/N (%) | | Intervention impact on past year physical IPV | Likelihood Ratio Test^±^ p-value |
| --- | --- | --- | --- | --- |
|  | Intervention arm | Control arm | aOR* (95%CI) |  |
|  | n/N (%) | n/N (%) |  |  |
| Overall study population | 113/550 (21%) | 117/575 (20%) | 0.98 (0.72 – 1.33) |  |
|  |  |  |  |  |
| Baseline report of lifetime physical IPV |  |  |  | p=0.701 |
| Never physical IPV | 20/230 (9%) | 21/250 (8%) | 1.08 (0.57 – 2.06) |  |
| Ever physical IPV | 93/320 (29%) | 96/325 (30%) | 0.94 (0.66 – 1.33) |  |
|  |  |  |  |  |
| Age |  |  |  | p=0.548 |
| Under 35 years | 79/319 (25%) | 82/332 (25%) | 0.91 (0.63 – 1.33) |  |
| 35+ years | 34/231 (15%) | 35/243 (14%) | 1.11 (0.66 – 1.89) |  |
|  |  |  |  |  |
| Education |  |  |  | p=0.768 |
| Primary or below | 94/444 (21%) | 94/450 (21%) | 1.00 (0.71 – 1.41) |  |
| Above primary | 19/106 (18%) | 23/125 (18%) | 0.89 (0.44 – 1.80) |  |
|  |  |  |  |  |
| Income |  |  |  | p=0.574 |
| Below median income | 72/367 (20%) | 67/339 (20%) | 0.93 (0.63 – 1.38) |  |
| Above median income | 41/183 (22%) | 50/236 (21%) | 1.12 (0.68 – 1.83) |  |
|  |  |  |  |  |
| Financial independence |  |  |  | p=0.475 |
| Probably/definitely couldn’t look after family on her income alone | 50/212 (24%) | 41/204 (20%) | 1.12 (0.69 – 1.84) |  |
| Probably/definitely could look after family on her income alone | 63/338 (19%) | 76/371 (20%) | 0.89 (0.60 – 1.32) |  |
|  |  |  |  |  |
| Baseline marital status |  |  |  | p=0.345 |
| Separated/divorced/widowed/ never married | 8/105 (8%) | 14/114 (12%) | 0.64 (0.25 – 1.63) |  |
| Currently married/living as married | 105/445 (24%) | 103/461 (22%) | 1.02 (0.74 – 1.42) |  |

*Estimated from mixed effects logistic regression models with random intercepts for group. Models included interaction term between intervention and sub-group characteristic, and fixed effects terms for age (linear), education (above primary/primary or below) and baseline past year physical IPV.

^±^Likelihood ratio test comparing model with and without interaction
