# Supplementary material for: Pathways to reduced physical intimate partner violence among women in north-western Tanzania: Evidence from two cluster randomised trials of the MAISHA intervention
Source: PLOS Glob Public Health. 2023 Nov 13;3(11):e0002497. doi: 10.1371/journal.pgph.0002497 (PMC10642778; doi:10.1371/journal.pgph.0002497)
Supplement: S1 Protocol — (DOCX) [file pgph.0002497.s007.docx]

**A cluster randomized controlled trial to assess the impact on intimate partner violence of a 10-session participatory gender training curriculum delivered to women taking part in a group-based microfinance loan scheme in Tanzania (MAISHA CRT01): study protocol**

Sheila Harvey, BSc (Hons), MSc, PhD *
Department of Global Health and Development, London School of Hygiene & Tropical Medicine, 15-17 Tavistock Place, London WC1H 9SH and Mwanza Intervention Trials Unit, PO Box 11936, Mwanza, Tanzania
[sheila.harvey@lshtm.ac.uk](mailto:sheila.harvey@lshtm.ac.uk)

Shelley Lees, MSc, MRes, PhD
Department of Global Health and Development, London School of Hygiene & Tropical Medicine, 15-17 Tavistock Place, London WC1H 9SH [shelley.lees@lshtm.ac.uk](mailto:shelley.lees@lshtm.ac.uk)

Gerry Mshana, PhD
National Institute for Medical Research, PO Box 1462, Mwanza, Tanzania
[gerrymshana@hotmail.com](mailto:gerrymshana@hotmail.com)

Daniel Pilger, MD, MSc
Mwanza Intervention Trials Unit, PO Box 11936, Mwanza, Tanzania
[Daniel.pilger@lshtm.ac.uk](mailto:Daniel.pilger@lshtm.ac.uk)

Christian Hansen, BSc, MSc, PhD
Department of Infectious Disease Epidemiology, London School of Hygiene & Tropical Medicine, Keppel Street, London WC1E 7HT and Mwanza Intervention Trials Unit, PO Box 11936, Mwanza, Tanzania
[christian.hansen@lshtm.ac.uk](mailto:christian.hansen@lshtm.ac.uk)

Saidi Kapiga, MD, MPH, ScD
Mwanza Intervention Trials Unit, PO Box 11936, Mwanza, Tanzania and
Department of Infectious Disease Epidemiology, London School of Hygiene & Tropical Medicine, Keppel Street, London WC1E 7HT
[saidi.kapiga@lshtm.ac.uk](mailto:saidi.kapiga@lshtm.ac.uk)

Charlotte Watts, PhD, FMedSci
Department of Global Health and Development, London School of Hygiene & Tropical Medicine, 15-17 Tavistock Place, London WC1H 9SH [charlotte.watts@lshtm.ac.uk](mailto:charlotte.watts@lshtm.ac.uk)

*corresponding author

**Citation for this paper:** Harvey, S., Lees, S., Mshana, G. *et al.* A cluster randomized controlled trial to assess the impact on intimate partner violence of a 10-session participatory gender training curriculum delivered to women taking part in a group-based microfinance loan scheme in Tanzania (MAISHA CRT01): study protocol. *BMC Women's Health* **18**, 55 (2018). https://doi.org/10.1186/s12905-018-0546-8

**ABSTRACT**

**Background:** Worldwide, almost one third (30%) of women who have been in a relationship have experienced physical and/or sexual violence from an intimate partner. Given the considerable negative impacts of intimate partner violence (IPV) on women’s physical health and well-being, there is an urgent need for rigorous evidence on violence prevention interventions.

**Methods:** the study, comprising a cluster randomized controlled trial (RCT) and in-depth qualitative study, will assess the impact on women’s past year experience of physical and/or sexual IPV of a participatory gender training curriculum (MAISHA curriculum) delivered to women participating in group-based microfinance in Tanzania. More broadly, the study aims to learn more about the factors that contribute to women’s vulnerability to violence and understand how the intervention impacts on the lives of women and their families. Sixty-six eligible microfinance loan groups are enrolled and randomly allocated to: the 10-session MAISHA curriculum, delivered over 20 weeks (n=33); or, to no intervention (n=33). Study participants are interviewed at baseline and at 24 months post-intervention about their: household; partner; income; health; attitudes and social norms; relationship (including experiences of different forms of violence); childhood; and community. For the qualitative study and process evaluation, focus group discussions are being conducted with study participants and MAISHA curriculum facilitators. In-depth interviews are being conducted with a purposive sample of 18 participants. The primary outcome, assessed at 24 months post-intervention, is a composite of women’s reported experience of physical and/or sexual IPV during the past 12 months. Secondary outcomes include: reported experience of physical, sexual and emotional/psychological IPV during the past 12 months, attitudes towards IPV and reported disclosure of IPV to others.

**Discussion:** the study forms part of a wider programme of research (MAISHA) that includes: a complementary cluster RCT evaluating the impact of delivering the MAISHA curriculum to women not receiving formal group-based microfinance; an economic evaluation; and a cross-sectional survey of men to explore male risk factors associated with IPV. MAISHA will generate rigorous evidence on violence prevention interventions, as well as further insights into the different forms and consequences of violence and drivers of violence perpetration.

**Trial registration:** ClinicalTrials.gov ID: NCT02592252, registered retrospectively on 13 August 2015

**Key words**

Maisha, intimate partner violence, cluster randomized controlled trial, qualitative, microfinance, gender training, violence prevention, Tanzania, Africa

**BACKGROUND**

Violence against women and girls is a major global public health and development concern. Empowering women and promoting gender equality is one of the 17 sustainable development goals outlined in the United Nations 2030 Agenda on Sustainable Development, which was adopted by countries in 2015. Ending all forms of discrimination against women and girls, including physical and sexual violence and other forms of abuse, is not only a human right issue but also crucial to accelerating sustainable development [1].

The past decade has seen a rapidly growing body of research on violence against women. Worldwide, almost one third (30%) of women who have been in a relationship have experienced physical and/or sexual violence by an intimate partner. The negative impacts of intimate partner violence (IPV) on women’s physical and mental health are considerable [2] and the impact on their children is of increasing concern, given that co-occurrence of exposure to IPV and other types of child maltreatment is high [3]. The World Health Organisation (WHO) has highlighted the urgent need for evidence on effective violence prevention interventions [4]. Although evidence is now starting to emerge, rigorous data on what works to prevent violence remain scarce. Data are highly skewed towards studies conducted in high-income countries with intervention research focused more on response than prevention [5].

One example of an intervention that aims to prevent women’s experience of IPV is the Intervention with Microfinance for AIDS & Gender Equity (IMAGE), which was developed in rural South Africa and combines group-based microfinance with a participatory gender and HIV training programme. In a cluster randomised controlled trial (RCT), IMAGE was shown, over a two-year period, to reduce women’s past year experience of physical and/or sexual IPV by 55% [6]. In addition, levels of household poverty were significantly reduced and participants were more empowered as evidenced by greater self-confidence, autonomy in decision making, and increased ability to challenge gender norms when compared with women in the control population [7]. These findings have led to national policy change and the formal inclusion of microfinance and the empowerment of women into the South African Government’s Strategic Plan for HIV/AIDS. Regional and international policy makers have asked whether, with appropriate national level refinement and adaptation, the IMAGE model would achieve the same level of impact if it was implemented in other sub-Saharan African settings.

High rates of IPV have been reported in Tanzania – the WHO multi-country study on women’s health and domestic violence found that almost 30% of ever-partnered women in a rural area of Tanzania had experienced physical and/or sexual violence from a partner in the year prior to the survey [8]. Ahead of setting up the MAISHA study to replicate the IMAGE study in Tanzania, a participatory social mapping study (unpublished) was conducted, comprising participatory group discussions and transect walks in a sample of neighborhoods in Mwanza city, northwestern Tanzania. The objectives of the study were to determine: 1) social and economic boundaries and activities in the study communities; 2) types and functioning of microfinance entities in the study communities; and, 3) feasibility of recruiting the required numbers of study participants and retaining them for over a year. In all the neighborhoods studied, both informal and formal microfinance activities were reported. Informal microfinance is initiated by neighbors (i.e. people who know each other) and involves small loans with no formal membership or loan records. Formal microfinance, delivered by developmental non-governmental organisations, requires registration with the organisation and involves relatively large loans with fixed interest rates. The social mapping study indicated that formal microfinance is not usually delivered to the poorest of the poor and that most women who take formal microfinance loans tend to come from households that are able to meet their basic daily needs and may even have accumulated some assets. It seems therefore, that women who do and do not engage in formal microfinance activities are probably different populations. Given this, the MAISHA study comprise two cluster RCTs to evaluate the impact of a participatory gender training curriculum on women’s past year experience of IPV. The first RCT (MAISHA CRT01), described in this paper, seeks to evaluate the impact of the curriculum delivered to women in established formal microfinance loan groups in Tanzania. The research question being addressed is: do women in established formal microfinance loan groups, who participate in a participatory gender training curriculum, experience lower levels of past-year IPV compared with women in established formal microfinance loan groups who do not? The study is being conducted in collaboration with the Bangladesh Rural Advancement Committee (BRAC), which is one of the leading microfinance providers in Mwanza and across Tanzania. The second RCT (MAISHA CRT02), described in a separate paper, seeks to evaluate the impact of the same curriculum delivered to women in newly-formed groups who are not engaged in formal group-based microfinance.

MAISHA is being implemented by the Tanzanian National Institute for Medical Research (NIMR), Mwanza Intervention Trials Unit (MITU) and London School of Hygiene & Tropical Medicine (LSHTM).

**Aim and objectives**

The overall aim of the study is to assess the impact on IPV of a participatory gender training curriculum (the MAISHA curriculum) delivered to women taking part in a formal group-based microfinance scheme. The primary objective is to assess the impact on women’s experience of physical and/or sexual IPV during the past 12 months. The secondary objectives are to assess the impact on:

- different forms of IPV – physical, sexual and emotional/psychological;
- women’s attitudes towards the acceptability of IPV; and
- women’s disclosure of violence to others.

The study also seeks, through an in-depth qualitative study, to:

- learn more about the factors that contribute to women’s vulnerability to violence; and
- to understand how the intervention impacts on the lives of participants and their families.

The theory of change model (Figure 1) maps out the key contextual factors that may influence the impact of the intervention, the components of the intervention, the expected initial, intermediate and longer-term outcomes of the intervention and the overall impact the intervention is designed to have on women in Tanzania.

**METHODS/DESIGN**

**Study design and setting**

This is a mixed methods study comprising a cluster RCT with a complementary in-depth qualitative study and an integrated process evaluation. The study is being conducted in Mwanza city, in northwestern Tanzania. In collaboration with BRAC, established microfinance loan groups in Mwanza city are being identified and assessed for eligibility to take part. Each member of a microfinance loan group is required to pay a deposit before receiving their first loan. They are also required to contribute a small payment each week as a social security deposit. The interest rate is fixed at 25%. The group meets every week to repay part of the loan with a maximum loan repayment time of six months. If an individual member of the group is unable to contribute her share of the loan repayment, the other members of the group must cover this.

Figure 1 Theory of Change Model


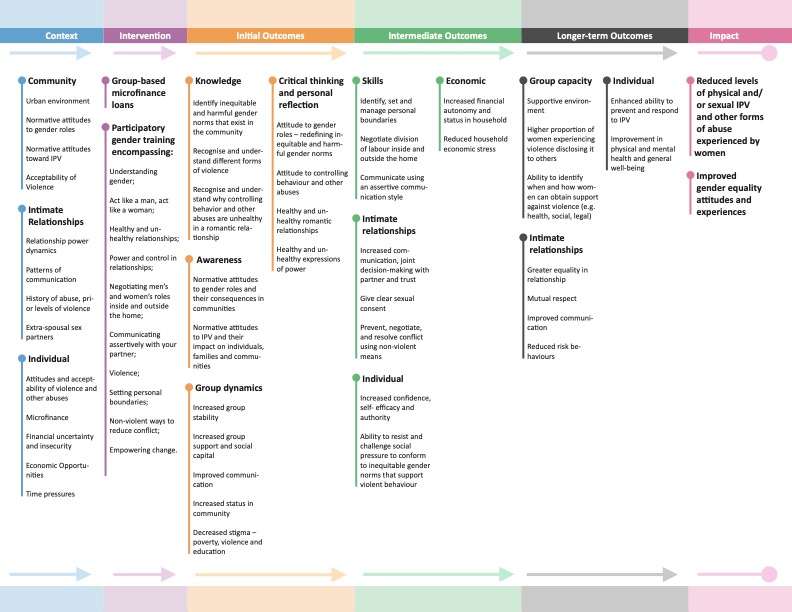


**Eligibility criteria**

Established microfinance loan groups that meet the following criteria are eligible for inclusion in the study:

1. there are less than 30 active members in the group;
2. there is a good attendance (repayment) record based on BRAC records; and
3. a minimum of 70% of active members consent to take part in the study, that is they:
   1. demonstrate comprehension of the study procedures;
   2. are willing to undergo the study procedures, including attending all 10 sessions of the MAISHA curriculum, if randomly assigned to this arm of the study; and
   3. have signed an informed consent form.

For each microfinance loan group enrolled, only women within the group who consent to take part, undergo study procedures.

**Intervention and comparator**

The group-based microfinance loans are delivered by BRAC with no involvement from the MAISHA study team. Microfinance groups allocated to the control arm continue to meet every week for loan repayments following BRAC procedures. Although the MAISHA team continues to keep in regular contact with the groups (to minimize losses to follow-up), there is no further intervention. Microfinance groups allocated to the intervention arm also continue to meet every week for loan repayments. In addition, on alternate weeks, either before or after the loan group meeting, they receive the MAISHA curriculum – *Wanawake na Maisha* (which means “women and life” in Swahili). The curriculum comprises 10 sessions and was developed for the MAISHA study, by EngenderHealth (an international non-profit organisation focussing on family planning, maternal health, HIV and AIDS and gender equity) in collaboration with LSHTM and MITU. Some of the curriculum activities for *Wanawake na Maisha* were adapted from other curricula [6, 9-13], including the *Sisters for Life* curriculum developed for IMAGE in South Africa [6]. The overall aim of the MAISHA curriculum is that, after completing the 10 sessions, participants will have developed skills to help them minimize, and potentially prevent, IPV within intimate relationships, as well as having increased capacity to defend themselves against IPV and the negative consequences resulting from IPV. The specific objectives of the curriculum are detailed in Table 1.

The MAISHA curriculum is delivered over 20 weeks. Each of the 10 sessions (outlined in Figure 1) is approximately an hour and a half to two hours giving a total time of approximately 20 hours. Each session is participatory and comprises: giving information to participants, small group activities and group discussions, and ending with a take home assignment designed to encourage participants to practice the skills covered during the session. The MAISHA curriculum is delivered by trained facilitators following the MAISHA curriculum manual, which provides detailed guidance for each session. The manual includes tips and notes for the facilitators, including examples of group ice-breakers and energisers. The facilitators have been trained by EngenderHealth to facilitate the MAISHA curriculum which included: gender equitable behavior and attitudes; managing group dynamics (including emotional reactions and disclosure of sensitive information); establishing a safe and comfortable learning environment; and encouraging all participants to take part in discussions. In addition, the training also included discussion around beliefs, including: the belief that intimate relationships should never be coercive, exploitative or abusive; belief in the importance of gender equity and women’s rights; and belief that inequitable gender norms can be changed.

Ongoing training of the MAISHA curriculum facilitators, including practicing facilitation skills through role play, is supported by MITU and LSHTM. The MAISHA curriculum facilitators are not involved in collection of baseline data or any outcome assessments for the study.

Table 1 Objectives of The MAISHA curriculum (*Wanawake Na Maisha*)

| **Objective number** | **Intended outcome for participants is that they should be able to:** |
| --- | --- |
| 1 | Identify inequitable and harmful gender norms that exist in their community, especially those norms that contribute to IPV |
| 2 | Explain how abiding to inequitable and harmful gender norms has health and social costs to women, men, families and the community |
| 3 | Re-define inequitable and harmful gender norms into equitable and healthy alternatives |
| 4 | Describe the characteristics of healthy and unhealthy romantic relationships |
| 5 | Explain why controlling and abusive behaviour is unhealthy in a romantic relationship |
| 6 | Explain healthy and unhealthy expressions of power |
| 7 | Identify, set and manage personal boundaries |
| 8 | Negotiate division of labour in and outside the home |
| 9 | Communicate using an assertive communication style |
| 10 | Identify different forms of violence including emotional, physical, economic and sexual |
| 11 | Explain the impact of intimate partner violence on the health and wellbeing of women, men, families and communities |
| 12 | Give clear sexual consent |
| 13 | Prevent, negotiate and resolve conflict using non-violent means |
| 14 | Resist and challenge social pressure to conform to inequitable gender norms that support violent behaviour |
| 15 | Identify when and how women can obtain support against violence (e.g. health, social, legal, etc.), if needed |

**Outcomes**

The primary outcome is a composite of women’s reported experience of physical and/or sexual IPV during the past 12 months and is assessed via a face-to-face interview at 24 months post-intervention (29 months post-randomization). The secondary outcomes, also assessed at 24 months post intervention, are women’s reported experience of specific forms of IPV during the past 12 months, as follows:

- physical IPV;
- sexual IPV; and
- emotional/psychological abuse.

Other secondary outcomes are:

- women’s attitudes towards the acceptability of IPV; and
- women’s disclosure of violence to others – for those who report physical and/or sexual IPV during the past 12 months.

Table 2 details the questions asked to assess the different forms of IPV, which have been adapted from the WHO Violence Against Women instrument [8].

**Participant timeline**

Following enrolment into the study, baseline data are collected from women who have consented to take part. Randomization occurs once all women in a block of six microfinance loan groups have completed the baseline interview. The intervention is delivered over 20 weeks (five months) and women in both study arms are then followed up 24 months later, i.e. 29 months post-randomization (Figure 2).

**Sample size**

The sample size calculation assumes an estimated prevalence of IPV during the past 12 months of 30% in the comparison arm, based on data from the WHO multi-country study in Tanzania [8]. A sample size of 33 microfinance loan groups per study arm with an average of 20 participants per group (allowing for 10% loss to follow-up) will provide 80% power to detect a reduction of 30% in physical and/or sexual IPV during the past 12 months, and 90% power to detect a reduction of 34%, assuming an intra-cluster correlation of 0.02. Even with an intra-cluster correlation of 0.04, the study will have 80% power to detect a reduction in IPV during the past 12 months of 33%.

Table 2 Questions used to assess different forms of intimate partner violence experienced by women taking part in the MAISHA study
(taken from the WHO Violence Against Women instrument [8]).

| **Type of violence** | **Questions** |
| --- | --- |
| **Physical violence** | **Has your current partner or any other partner ever:** |
|  | 1. Slapped you or thrown something at you that could hurt you? |
|  | 2. Pushed you or shoved you or pulled your hair? |
|  | 3. Hit you with his fist or with something else that could hurt you? |
|  | 4. Kicked you, dragged you or beaten you up? |
|  | 5. Choked or burnt you on purpose? |
|  | 6. Threatened to use or actually used a gun, knife or other weapon against you? |
|  |  |
| **Sexual violence** | **Have you ever had sexual intercourse with your current partner or any other partner:** |
|  | 1. After he forced you by threatening you, holding you down or hurting you in some way? |
|  | 2. When you did not want to because you were afraid that your partner would hurt you or someone you cared about if you refused? |
|  | 3. When you did not want to because you were afraid that your partner would leave you or take another girlfriend if you refused? |
|  |  |
| **Controlling behavior** | **Thinking about your (current or most recent/past) partner, would you say it is generally true that he:** |
|  |  |
|  | 1. Tries to keep you from seeing your friends? |
|  | 2. Tries to restrict contact with your family of birth? |
|  | 3. Insists on knowing where you are at all times? |
|  | 4. Is jealous and gets angry if you speak with another man? |
|  | 5. Is often suspicious that you are unfaithful? |
|  |  |
| **Economic abuse** | **Thinking about your (current or most recent/past) partner, would you say it is generally true that he:** |
|  | 1. Refuses to give you enough money for household expenses, even when he has money for other things? |
|  | 2. Takes money that you have earned away from you? |
|  | 3. Makes important financial decisions without consulting you? |
|  |  |
| **Emotional abuse** | **Has your current partner, or any other partner ever:** |
|  | 1. Insulted you or made you feel bad about yourself? |
|  | 2. Belittled or humiliated you in front of other people? |
|  | 3. Done things to scare or intimidate you on purpose (e.g. by the way he looked at you, by yelling and smashing things)? |
|  | 4. Verbally threatened to hurt you or someone you care about? |

For each type of violence/abuse, if a woman answers yes to one of more of the questions, then she is recorded has having experienced that form of violence/abuse.

A woman is recorded as having experienced physical *and/or* sexual violence (primary outcome) if she answers yes to one or more of the six questions relating to physical violence *and/or* one or more of the three questions relating to sexual violence.

Figure 2 Overview of Participant Flow

**ASSESSMENT OF ELIGIBILITY AND RECRUITMENT**

Existing microfinance loan groups (n=66)

≥70% members consent

Following informed consent,

complete baseline interview

**RANDOMISATION**

In blocks of six groups

Microfinance loans

+

participatory gender training

(n=33 groups)

Microfinance loans

only

(n=33 groups)

**FOLLOW-UP – 29 MONTHS POST-RANDOMISATION**

Assess primary and secondary outcomes

**Recruitment of microfinance groups**

The study team, in collaboration with BRAC, has identified three neighborhood BRAC branches, out of the seven branches operating across Mwanza city, in which to recruit established microfinance loan groups. Within these three neighborhoods there are 220 established microfinance loan groups. The study team works closely with BRAC to select groups to approach and invite to take part in the study. Selection of groups to approach is based on factors such as the length of time the group has been established (at least one year), the size of the group (between 15 and 30 active members), and good attendance at week loan meetings with a good record of loan repayments.

**Allocation method and blinding**

Randomization occurs in blocks of six microfinance loan groups. To ensure transparency of the process to communities, randomization and allocation is a participatory process involving the study team and a representative from each of the six microfinance loan groups to be randomized. Groups are allocated to either intervention or control by tossing a coin. First, representatives from each of the six microfinance groups are randomly divided into two sets (A and B) of three groups. This is done by each representative drawing a folded sheet of paper (with A or B written on it) from a box. One of the representative is asked to call (heads or tails) for her set of three groups to be allocated to the intervention. A study team member then tosses the coin. Given the nature of the intervention, it is not possible to blind participants, or the study team involved in day-to-day operations and delivery of the MAISHA curriculum, after assignment of the intervention. Data analysts will be blinded to allocation.

Table 3 MAISHA Study Schedule (based on SPIRIT template [14])

|  | **STUDY PERIOD** | | | | | | | |
| --- | --- | --- | --- | --- | --- | --- | --- | --- |
|  | **Enrolment** | **Allocation** | **Intervention** | | | | | **Closeout** |
| Time point (months) | ***- M1*** | ***0*** | ***M1*** | ***M2*** | ***M3*** | ***M4*** | ***M5*** | ***M29*** |
| **Enrolment** |  |  |  |  |  |  |  |  |
| Eligibility screen | x |  |  |  |  |  |  |  |
| Informed consent | x |  |  |  |  |  |  |  |
| **Allocation** |  | x |  |  |  |  |  |  |
| **Interventions** |  |  |  |  |  |  |  |  |
| MF only (control) |  |  | x | x | x | x | x |  |
| MF & PGT (intervention) |  |  | x | x | x | x | x |  |
| **Assessments** |  |  |  |  |  |  |  |  |
| ***Baseline:*** |  |  |  |  |  |  |  |  |
| Socio-demographics | x |  |  |  |  |  |  |  |
| Physical IPV ^a^ | x |  |  |  |  |  |  |  |
| Sexual IPV ^a^ | x |  |  |  |  |  |  |  |
| Emotional abuse ^a^ | x |  |  |  |  |  |  |  |
| Attitudes about IPV | x |  |  |  |  |  |  |  |
| Disclosure of IPV to others ^a^ | x |  |  |  |  |  |  |  |
| In-depth interview ^b^ |  | x |  |  |  |  |  |  |
| Focus group discussion ^b^ |  | x |  |  |  |  |  |  |
| ***Post-intervention:*** ^c^ |  |  |  |  |  |  |  |  |
| In-depth interview ^b^ |  |  |  |  |  |  | x |  |
| Focus group discussion ^b^ |  |  |  |  |  |  | x |  |
| ***Follow-up:*** |  |  |  |  |  |  |  |  |
| Socio-demographics |  |  |  |  |  |  |  | x |
| Physical IPV ^a^ |  |  |  |  |  |  |  | x |
| Sexual IPV ^a^ |  |  |  |  |  |  |  | x |
| Emotional abuse ^a^ |  |  |  |  |  |  |  | x |
| Attitudes about IPV |  |  |  |  |  |  |  | x |
| Disclosure of IPV to others ^a^ |  |  |  |  |  |  |  | x |
| In-depth interview ^b^ |  |  |  |  |  |  |  | x |
| Focus group discussion ^b^ |  |  |  |  |  |  |  | x |

MF-group based microfinance loan; PGT-participatory gender training; IPV-intimate partner violence;

^a^ Reported experience in past 12 months

^b^ Participants will be random sample of women from control and intervention arms – same women will participate at three time-points

^c^ immediately following completion of the MAISHA curriculum

**Data collection methods – quantitative**

The MAISHA study schedule is outlined in Figure 3 (adapted from the SPIRIT template [14]). Data are collected at the following time points:

1. Baseline (prior to randomization) – following informed consent procedures, a face-to-face interview is conducted using a structured questionnaire adapted from the WHO Violence Against Women instrument [8]. The MAISHA questionnaire has seven sections which ask the woman about her: household; partner; income; health; attitudes and social norms; relationship (including experiences of violence); childhood; and about her community. The questionnaire has been translated into Swahili (the national language) and interviews are conducted in private by female interviewers trained in interviewing techniques, gender issues, violence and ethical issues related to research on IPV [15].

2. Intervention – during the 20-week intervention period, the following data are collected: attendance, or not, at the MAISHA curriculum sessions – to understand the “dose” of intervention received; and reasons for non-attendance at the MAISHA curriculum sessions – to understand the potential barriers to attendance.

3. 29 months post-randomization – a face-to-face interview is conducted using a structured questionnaire similar to that used at baseline and following the same procedures.

**Data collection methods – qualitative**

A total of 54 in-depth interviews (IDIs) are being conducted with participants. Eighteen women are being purposefully selected from the two study arms to represent women who do and do not report IPV at baseline. A separate team of trained interviewers conduct the IDIs and are blinded as to whether, or not, a woman has reported IPV. Each woman is invited to attend three IDIs – pre-intervention, immediately post-intervention and 24 months post-intervention. The IDIs explore the participants’ life stories and experiences of microfinance, the socio-cultural and structural factors associated with IPV and personal experiences of IPV and its impact on both themselves and their children. For women in the intervention arm, the post-intervention IDIs also explore their views and experiences of the MAISHA curriculum and its impact on their experiences of IPV. Five trial participants from the intervention arm who drop out of the MAISHA curriculum after attending two sessions will be invited to participate in an IDI to explore their reasons for withdrawal from the MAISHA curriculum.

Up to 10 key informant interviews are being conducted with local government and non-governmental organization officials, police, influential community leaders (e.g. religious leaders) and health care professionals. Interviews are conducted pre-intervention and 24 months post-intervention and explore the wider social and political context for IPV.

Twenty-seven focus group discussions (FGDs) are being conducted – comprising nine FGDs at three time points (pre-intervention, immediately post-intervention and 24 months post-intervention). Six FGDs are being conducted with women in the intervention arm and three with women in the control arm. Where possible the same women (approximately 10 per focus group) are asked to attend at all three time points. The FGDs explore experiences of microfinance, the socio-cultural and structural factors associated with IPV. The post-intervention FGDs with women in the intervention arm also explore their views and experiences of the MAISHA curriculum and its impact on their views of IPV.

FGDs are being conducted with the MAISHA curriculum facilitators to explore their views on the curriculum as a whole and on specific modules, the challenges they have experienced when delivering the sessions, and their perspectives on the impact of the MAISHA curriculum.

The photo voices method is being used to enhance understanding of IPV and intimate relationships. A total of nine women (six from the intervention arm and three from the control arm) are invited to take part immediately post-intervention. Participants receive two days training on using a camera and the ethics of taking photographs in the community before being asked to spend one week photographing everyday lives in their community with a focus on healthy relationships. The participants are then interviewed and asked to provide oral narratives of the photographs they have taken.

Participatory observations are being conducted at selected microfinance loan group meetings and at the MAISHA curriculum sessions, ensuring that each session is observed at least once. Social scientists have Informal conversations with study participants to assess their impressions of the curriculum sessions and its immediate impact.

**Data management**

Questionnaire data collected from study participants at baseline and at 29 months post-randomization are recorded directly onto a tablet computer. The questionnaire forms have in-built checks to minimize the level of missing data and to minimize entry of erroneous data. The data recorded on the tablet computer are uploaded to the study database daily and checked for missing and/or erroneous data. Any data queries are sent to the team leader to be resolved with the research assistants conducting the interviews.

Attendance at the MAISHA curriculum sessions and reasons for non-attendance are recorded on paper and entered into the study database following double-entry data procedures. Data are checked for missing and/or erroneous data. Any data queries are sent to the team leader to be resolved with the MAISHA curriculum facilitators.

All IDIs and FGDs are recorded with the participant’s consent. Hand written notes are taken during the participatory observations of the MAISHA curriculum sessions and microfinance loan group meetings. Audio recordings and hand written notes are transcribed and translated from Swahili (the national language) into English. A sample of the transcripts are checked for quality of transcription and translation. Transcripts are imported to the qualitative analysis package NVIVO (QSR International Pty Ltd, Doncaster, Australia). All visual material, including photographs from the photo voices activities, are imported into the same package.

All study data are stored in secure databases with restricted access. Each participant is allocated a unique study identifier. Names and other identifiers are not recorded in the study database. Paper records – e.g. consent forms, tracking forms with names and contact details – are stored securely in locked filing cabinets in secure offices within the study coordinating center at MITU, which has 24-hour security and restricted access.

**Statistical methods**

A detailed statistical analysis plan will be prepared prior to follow-up interviews. Data from the baseline interviews will be used to verify the sample size calculations and to identify differences between clusters. The coefficient of variation across clusters will be calculated based on the reported prevalence of IPV. Data from the baseline quantitative interviews will also be used to identify important predictors for IPV and important health-related outcomes, such as poor mental health.

The primary study analysis will adopt an intention to treat approach, assessing the impact of the intervention on women in the intervention arm at 29 months post-randomization (24 months post-intervention), irrespective of whether or not they received the full “dose” (i.e. 10 sessions) of the MAISHA curriculum. Secondary analyses will be conducted to investigate differences in impact according to the dose of the intervention received.

The primary outcome variable (reported experience of a composite of physical and/or sexual IPV during the past 12 months) will be analyzed in a random intercepts logistic regression model to account for the clustered study design, and adjusted for differences in baseline characteristics where relevant. The analysis will be repeated to examine the secondary outcome variables – reported experience of physical IPV, sexual IPV and emotional/psychological abuse during the past 12 months, attitudes towards the acceptability of IPV and, disclosure of violence to others among women who report having experienced physical and/or sexual IPV during the past 12 months. Multiple imputation will be used to simulate missing outcome data. The imputation model will be informed by empirical patterns in the IPV data at baseline and at follow-up. A sensitivity analysis will be conducted, excluding women who participated in the qualitative sub-study (including IDIs, FGDs and photo voices) on the basis that the additional contact of this sub-sample with the study team, as part of these activities, may impact on the effect of the intervention. The analysis will assess if there is any change in the magnitude of the effect.

Steps have been taken to minimize contamination of the control arm which includes recording women’s attendance at sessions. The potential for direct and indirect contamination of control arm women will be investigated by asking women during follow-up if they attended any of the MAISHA curriculum sessions or if they have discussed any of the sessions with women in the intervention arm.

**Safety monitoring**

Given that no outcome data (i.e. experiences of IPV) are collected during the five-month intervention period or during the period up to 24 months post-intervention, a data monitoring committee has not been established as no interim analyses are planned. The study is being conducted following the WHO’s guidelines on researching violence against women [15]. Female interviewers for the quantitative baseline and follow-up interviews and for the qualitative IDIs have received training in interviewing techniques, gender issues, violence and ethical issues related to research on IPV. It is anticipated that any harm to women as a result of taking part in the study will be minimal. All participants are provided with information about organizations offering support to women (and their children, if appropriate) experiencing violence and other forms of abuse. Participants who report violence and other forms of abuse are offered counseling by a trained member of the study team and referral to an appropriate organization for ongoing support.

**Auditing**

Regular audits of the conduct of the study are carried out by members of the study team. These include checks that participant informed consent procedures have been followed correctly, observation and assessments of facilitation of the MAISHA curriculum sessions, and monitoring of participant attendance at MAISHA curriculum sessions and follow-up of non-attenders.

**Informed consent**

Once a microfinance loan group is identified as meeting eligibility, the study team attends the weekly meetings to present information about the study and provides a copy of the participant information sheet (see: Additional File 1) to each of the microfinance loan group members. Each microfinance loan group member meets with a member of the study team to go through the participant information sheet in detail and to allow the microfinance loan group member to ask questions about the study. If the woman agrees to participate and has demonstrated that she understands the study procedures, she is invited to sign the consent form (see: Additional File 1). Participants and key informants who are invited to take part in IDIs are given a participant information sheet providing information about the IDI (see: Additional File 2). A member of the study team meets with the participant/key informant to go through the participant information sheet in detail and to allow the participant to ask any questions. If the participant/key informant agrees to participate in the IDI she/he is invited to sign a consent form (see: Additional File 2). Participants who are invited to take part in an FGD are given an information sheet about the FGD (see: Additional File 3) following the same procedures described above for obtaining informed consent.

**Confidentiality**

Participants’ names and any information that could identify them is kept confidential. Women are allocated a unique study number. The questionnaires for the quantitative baseline and follow-up interviews are anonymous and responses to questions are entered directly onto a tablet computer. On the same day as the interview, data are uploaded to the secure study database and removed from the tablet computer before the next interview is conducted. Qualitative IDIs are audio recorded with the participant’s consent. The recordings are labelled with the study number only and are destroyed once the recording has been transcribed and translated to English. All identifiers will be destroyed at the end of the study.

**Ancillary and post-trial care**

During the 24-month follow-up period following delivery of the intervention, the study team maintains regular contact with participants in order to minimize losses to follow-up. Women who report violence and other forms of abuse during this time are offered support and referred to appropriate organizations for ongoing support post-study.

**Protocol amendments**

Since the start of recruitment, there has been one amendment to the protocol approved by the ethics committees. The follow-up period has been extended from 12 months post-intervention to 24 months post-intervention following confirmation of the additional funding required. The study investigators felt that this would be a more appropriate time point at which to assess the effectiveness of the MAISHA curriculum in reducing women’s experience of IPV, and to ensure greater comparability with the IMAGE study. In addition, the secondary outcomes were reviewed and amended to ensure that they were clearly defined, specific and measurable.

**Dissemination policy**

The study findings will be widely disseminated through both formal and informal mechanisms. Meetings will be held with participants to inform them of the results of the study. For women in the control arm, information will be provided as to how the MAISHA curriculum will be expanded into their communities, if it is shown to impact on levels of IPV. The study findings will be presented to key stakeholders at local, regional and national level in Tanzania and at relevant regional, national and international conferences and meetings. Reports of the study will be prepared by the study team for submission to peer-review scientific journals. Other strategies to facilitate dissemination of the results of the study will be developed through collaboration with organisations, consortia and forums such as the STRIVE Research Programme Consortium (Tackling the structural drivers of HIV) and the Sexual Violence Research Initiative (SVRI).

**DISCUSSION**

The cluster RCT described in this paper (MAISHA CRT01) forms part of the MAISHA study, a programme of research that also includes: a second complementary cluster RCT (MAISHA CRT02) to evaluate the impact of the MAISHA curriculum delivered to newly-formed groups of women who are not engaged in formal group-based microfinance activties; an economic evaluation to evaluate the total costs of the development and implementation of MAISHA CRT01 and MAISHA CRT02; and a cross-sectional survey of the male partners of women taking part in CRT01 to identify risk factors in men associated with IPV (e.g. alcohol use, employment and abuse during childhood) and to explore whether the intervention delivered to women has impacted on their male partners’ attitudes and behavior.

**Strengths and limitations**

A major strength of MAISHA CRT01 is its mixed methods design, utilizing both qualitative and quantitative approaches, to better understand the effects of the intervention and how it is experienced by the participants [16]. Utilizing a randomized design will ensure scientific rigor in the quantitative evaluation of the intervention. Another strength of the study is the large sample size (66 established microfinance loan groups), which represents around one third of the established microfinance loan groups within the defined study area. Although it is possible that these groups may not be a representative sample of all established microfinance loan groups in Mwanza city. The study does not have the resources to collect data on the characteristics of women in non-participating microfinance loan groups to assess the extent of any selection bias. However, it is important to note that any such bias would affect how generalizable the results of the study are to the wider population of women engaged in formal group-based microfinance activities, rather than compromise the internal validity of the study itself. Another limitation, common to studies of complex interventions, is that it will be difficult to unpack which elements of the intervention may or may not have an impact on IPV. An integral part of the MAISHA intervention is that it enables women in microfinance loan groups to have more time together and thereby more time for interaction. Women in the intervention groups meet for longer (either before or after their loan group meeting) on alternate weeks, over a 20-week period, in order to complete the MAISHA curriculum. Whereas, women in the control groups continue with their usual weekly loan group meetings with no additional time for interaction. If an impact on IPV rates is observed in the intervention groups, it may be difficult to determine whether it has resulted from the additional group time or the curriculum, or a combination of both. Data from the complementary qualitative study will be invaluable in exploring women’s experiences of the MAISHA curriculum and format, potential reasons for its success or failure to prevent IPV and variations in impact across groups and/or individual participants.

**Progress and timelines**

For MAISHA CRT01, recruitment of 66 established formal microfinance loan groups is complete. Of these, 33 groups were randomly allocated to the intervention arm and 33 groups to the control arm. Delivery of the MAISHA curriculum to the 33 groups allocated to the intervention arm is complete. Baseline interviews with participants indicate a prevalence of physical and/or sexual IPV during the past 12 months of 27% (95% confidence interval: 24% to 29%) [17], confirming the assumption made for the sample size calculation of 30% prevalence of physical and/or sexual IPV during the past 12 months. Follow-up of participants for assessment of the primary and secondary outcomes at 24 months post-intervention is almost complete. Data analysis will be conducted from 2018 onwards and the results of the trial disseminated as described above.

For MAISHA CRT02, formation and recruitment of 66 groups of women not engaged in formal group-based microfinance activities is complete. Delivery of the MAISHA curriculum to the 33 groups allocated to the intervention arm is also complete. Follow-up of women for the primary and secondary outcomes will commence in 2018. A separate paper describing the protocol for MAISHA CRT02 has been prepared.

**Secondary analyses of the MAISHA study datasets**

The different components of the MAISHA study will generate a large volume of quantitative and qualitative data on the prevalence of IPV in Mwanza (Tanzania’s second city), risk factors for IPV, the impact of interventions to prevent women’s experience of IPV, attitudes towards the acceptability of IPV and the socio-cultural and structural factors associated with IPV. Secondary analyses of these data are planned, which will include analyses to explore and better understand how the MAISHA curriculum may or may not impact on: the different forms of IPV; patterns of IPV; patterns of communication between couples; and women’s physical and mental health, including sexual behavior. In addition, using data collected from the male partners of CRT01 participants, analyses will explore men’s knowledge and attitudes towards IPV and how these compare with those of women, and whether the MAISHA curriculum delivered to women has any impact on their male partners.

Based on data collected at baseline and at follow-up, analyses will be conducted to explore changes over time: in patterns of IPV experienced by women; in women’s attitudes towards the acceptability of IPV; and, changes in women’s physical and mental health. Structural equation modelling techniques and factor analysis, where relevant, will be used to investigate the hypothesized pathways of IPV, and to verify the pre-conceived theory of change model. Analyses of the qualitative data will include exploration of: the social and political context for IPV; socio-cultural and structural factors associated with IPV; and women’s experiences and views on interventions to prevent IPV.

**Conclusion**

In summary, the MAISHA study aims to address the urgent need for rigorous evidence on violence prevention interventions, the need for more data on the different forms of violence, the need to better understand the consequences of violence, such as the impact on the health of women and their families, and the need to better understand the drivers of violence perpetration.

**DECLARATIONS**

**Abbreviations**

BRAC – Bangladesh Rural Advancement Committee

DFID – Department for International Development

FGD – focus group discussion

IDI – in-depth interview

IMAGE – Intervention with Microfinance for AIDS & Gender Equity

IPV – intimate partner violence

LSHTM – London School of Hygiene & Tropical Medicine

NIMR – National Institute for Medical Research

MITU – Mwanza Intervention Trials Unit

RCT – randomized controlled trial

SPIRIT – Standard Protocol Items: Recommended for Intervention Trials

SVRI – Sexual Violence Research Initiative

WHO – World Health Organization

**Ethics approval and consent to participate**

The MAISHA study is being conducted following WHO recommendations on researching violence against women [15]. It has been approved by the Tanzanian National Health Research Ethics Committee of the National Institute for Medical Research (Ref: NIMR/HQ/R.8a/Vol. IX/1512), and the ethics committee of the London School of Hygiene & Tropical Medicine (Ref: 11642). The study is implemented in close collaboration with local leaders and a community liaison system has been set up to facilitate the study team working with the communities where the study operates.

Written informed consent (witnessed by an independent witness for illiterate participants) is obtained from all participants before administering any study procedures.

The study sponsor is the London School of Hygiene & Tropical Medicine (Sponsor reference: QA430)

**Availability of data and material**

The MAISHA study datasets are not publicly available at this point as the study is ongoing. Once the main study analyses are complete, data will be made available upon request and in accordance with NIMR and LSHTM regulations.

**Competing interests**

Following initiation of the study, Professor Watts has been seconded to DFID as their chief scientific advisor. Her ongoing role in this study is in her academic capacity at LSHTM.

No other interests declared.

**Funding**

MAISHA is supported by the STRIVE Research Programme Consortium funded by UK Aid from the Department for International Development (DFID) and another donor, who wishes to remain anonymous. The views expressed in this paper do not necessarily reflect the Department’s official policies.

The funding bodies and sponsor have had no role in the design of the study or in writing this manuscript, and will not have any role in its conduct, analyses and interpretation of data, or decisions to disseminate the results.

**Authors’ contributions**

CW, SK and SL designed the study and led the grant application. SH and SK provide methodological input and oversee the conduct of the study and management of the research teams. DP previously provided methodological input and coordinated day-to-day management of the study. GM and SL provide support for the social science aspects of the study. CH provides statistical and methodological input. All authors contributed to the manuscript preparation, and approved the final manuscript for submission.

**Acknowledgements**

First and foremost, we wish to thank all study participants for their time and commitment to the study. We are also grateful to the MAISHA study team and the support staff at MITU for their hard work and dedication.

**REFERENCES**

1. United Nations Sustainable Development Goals. <http://www.undp.org/content/undp/en/home/sdgoverview/post-2015-development-agenda.html>. Accessed 22 February 2018
2. World Health Organization, London School of Hygiene & Tropical Medicine, South African Medical Research Council. Global and regional estimates of violence against women: prevalence and health effects of intimate partner violence and non-partner sexual violence. Geneva, World Health Organization 2013. <http://apps.who.int/iris/bitstream/10665/85239/1/9789241564625_eng.pdf>. Accessed 22 February 2018.
3. Wathen CN, Macmillan HL. Children’s exposure to intimate partner violence: impacts and interventions. Paediatr Child Health 2013;18(8):419-422.
4. World Health Organization and London School of Hygiene and Tropical Medicine. Preventing intimate partner and sexual violence against women: Taking action and generating evidence. Geneva, World Health Organization 2010. <http://www.who.int/violence_injury_prevention/publications/violence/9789241564007_eng.pdf>. Accessed 22 February 2018.
5. Elsberg M, Arango DJ, Morton M, Gennari F, Kiplesund S, Contreras M, Watts C. Prevention of violence against women and girls: what does the evidence say? Lancet 2015;385(9977):1555-66.
6. Pronyk PM, Hargreaves JR, Kim JC, Morison LA, Phetla G, Watts C, Busza J, Porter JD: Effect of a structural intervention for the prevention of intimate-partner violence and HIV in rural South Africa: a cluster randomised trial. Lancet 2006;368(9551):1973-1983.
7. Kim J, Ferrari G, Abramsky T, Watts C, Hargreaves J, Morison L, et al. Assessing the incremental effects of combining economic and health interventions: the IMAGE study in South Africa. Bull World Health Organ. 2009; 87(11): 824-32.
8. Garcia-Moreno C, Jansen H, Ellsberg M, Heise L, Watts C. Prevalence of intimate partner violence: findings from the WHO multi-country study on women's health and domestic violence. Lancet 2006;368(9543):1260-9*.*
9. The ACQUIRE Project, EngenderHealth, Promudo. Engaging Boys and Men in Gender Transformation: The Group Education Manual. New York, 2008. <http://www.acquireproject.org/archive/files/7.0_engage_men_as_partners/7.2_resources/7.2.3_tools/Group_Education_Manual_final.pdf>. Accessed 22 February 2018.
10. EngenderHealth. CoupleConnect: A Gender-Transformative HIV Prevention Curriculum for Tanzanian Couples. CHAMPION Brief No 3, May 2014.

<https://www.engenderhealth.org/files/pubs/project/champion/CHAMPION-Brief-3-CoupleConnect_lowres.pdf>. Accessed 22 February 2018.

1. Levack A, Rolleri L, DeAtley J. Gen.M: A Gender Transformative Teenage Pregnancy Prevention Curriculum. New York: EngenderHealth; 2014.
2. Raising Voices. SASA!. [*http://raisingvoices.org/sasa/*](http://raisingvoices.org/sasa/). Accessed 22 February 2018.
3. Population Council. It’s All One Curriculum: Guidelines and Activities for Unified Approach to Sexuality, Gender, HIV, and Human Rights Education [*http://www.popcouncil.org/research/its-all-one-curriculum-guidelines-and-activities-for-a-unified-approach-to-*](http://www.popcouncil.org/research/its-all-one-curriculum-guidelines-and-activities-for-a-unified-approach-to-). Accessed 09 January 2018 .
4. Chan AW, Tetzlaff JM, Gotzsche PC et al. SPIRIT 2013 explanation and elaboration: guidance for protocols of clinical trials. BMJ 2013;346:e7586.
5. Ellsberg M, and Heise L. Researching Violence Against Women: A

Practical Guide for Researchers and Activists. Washington DC, United States: World Health Organization, PATH; 2005.

1. Lewin S, Glenton C, Ozman AD. Use of qualitative methods alongside randomized controlled trials of complex healthcare interventions: methodological study. BMJ 2009;339:b3496.
2. Kapiga S, Harvey SE, Muhammad AK, Stoeckl H, Mshana G, Hashim R, Hansen C, Lees S, Watts C. Prevalence of intimate partner violence and abuse and associated factors among women enrolled into a cluster randomized controlled trial in northwestern Tanzania. BMC Public Health 2017;17:190.

ADDITIONAL FILES

Additional File 1

Title: Participant Information and Consent Form for MAISHA CRT01

Description: Information provided to potential participants, as part of the informed consent process for the MAISHA study, and the informed consent form signed by participants who agree to take part in the study.

Additional File 2

Title: Participant Information and Consent Form for MAISHA CRT01 – In-depth Interview

Description: Information provided to potential participants, as part of the informed consent process for participant and key informant in-depth interviews, and the informed consent form signed by participants and key informants who agree to take part in the in-depth interviews.

Additional File 3

Title: Participant Information and Consent Form for MAISHA CRT01 – Focus Group Discussion

Description: Information provided to potential participants, as part of the informed consent process for focus group discussion, and the informed consent form signed by participants who agree to take part in the focus group discussions.

**ADDITIONAL FILE 1**

**A cluster randomised controlled trial to assess the incremental impact on intimate partner violence of adding a 10-session participatory gender training programme to an existing microfinance intervention for women in Tanzania (MAISHA CRT01): study protocol**

**PARTICIPANT INFORMATION AND CONSENT FORM FOR MAISHA CRT01**

Version 1.2, 23 Sep 2014

**Introduction**

We are conducting research on an important issue related to health and healthy relationships. We would like to find out if you are interested to participate in this project. This research is being conducted by the National Institute for Medical Research (NIMR), Mwanza centre, and the Mwanza Intervention Trials Unit (MITU) in collaboration with the London School of Hygiene & Tropical Medicine (LSHTM). We have been approved to conduct this research by the Tanzania Ministry of Health and Social Welfare and by the London School of Hygiene & Tropical Medical ethics committee.

This form provides information about the study procedures. After reading and talking about the information provided with the study staff, you will be able to decide whether you want to take part in this study. If you decide to take part, we will ask you to sign this consent form.

Please note that:

- Your decision to take part is entirely voluntary. It is completely up to you to decide whether to take part in this project.
- You may decide not to take part, and not lose any of your rights or benefits such as the standard medical care you usually receive.
- If you decide to take part, you may drop out at any time, for any reason, without losing any rights or benefits.

**What is the purpose of the study?**

The purpose of this study is to explore ways to improve relationships and health more generally. In this study, a number of women groups receiving small financial loans, using a local organization called BRAC, will be selected and invited to take part in the study. Once the groups join the study, they will be divided without following any specific order (i.e. by chance), to either receive training on gender issues using methods which allow active participation during the training sessions, or not receiving such training.

After two years of implementing these activities, we will assess the impact on relationships for the women in the study as well as other health related outcomes. We will also measure changes on the ability of women to have control over their everyday lives, and the economic and health benefits. We will also assess the cost of implementing these activities against their effectiveness.

**RESEARCH PROCEDURES**

If you decide to participate, you will be asked to take part in the following study procedures. You are free to withdraw from any of these procedures at any time:

1. Preparation group meetings

As part of the study, you have been asked to attend four group meetings with up to 30 other women. At the first meeting, you will be provided with an explanation of the study, and you will receive this form and research staff will go through it with you and explain the study procedures in detail. During the meeting, you will have time to ask any questions you might have from reading this form. Every group that takes part in this study has the chance to be allocated to take part in the activities discussed above.

Please note all those who agree to participate in this study will be interviewed at the beginning and towards the end of the study. Participation by your group in any of the activities will be determined by chance. This means that your group will have equal chance of taking part in any of the activities described under (2) below.

2. Group activities

The group activities you could be participating in are:

- If your group is selected to receive training on gender, your group will be provided with 10 training sessions covering important issues such as the role of women in your community, (domestic) violence, HIV and other health related issues at a place and time convenient to all the group members. This could be after your weekly BRAC loan group meetings or at a different time. The sessions will be conducted over a period of up to 6 months. These sessions will be led by an experienced facilitator and each session will last about two hours. All women in these groups will be asked to attend an interview before and after the training sessions.

OR

- If your group is not selected to receive training on gender, you will only be asked to attend two interviews at the beginning of the study and towards the end (first interview and second interview).

3. First Interview

After you have agreed to take part in the study, a researcher will visit you at an agreed location and conduct an interview. You will be asked a series of questions about yourself, your health and your personal experiences in relationships.

This interview will be held by a trained interviewer and at a place convenient to you. Everything you tell us will be kept confidential. At any time during the interview you can refuse to answer questions or withdraw from the interview. As the interview will take between two and three hours to complete, you may also ask for a break if you are tired or something requires your immediate attention. This interview is very important for this study and if you agree to participate in this study you also agree to be interviewed.

4. Second interview

After your group has completed the assigned activity, you will be contacted a year later to be interviewed for a second time. Similar to the first interview, you will be asked a series of questions about the same topics. The purpose of this interview is to see if attending the group activities has resulted in any changes in your life. As for the first interview, this interview will be conducted at a place convenient to you and all your answers will be kept confidential and you may refuse to answer if you are not comfortable with some questions. This second interview will also take about two to three hours to complete. Again, this interview is very important and if you agree to take part in this study you also agree to this second interview.

5. Other interviews

You may be asked at any time during the study to attend an individual or group interview or other activities such as mapping your community or making a photographic record of your community in order to better understand your knowledge and perceptions of healthy relationships and what you think about the activities you are taking part in. If you are invited to attend other interviews you will be provided with a full explanation and you will sign a separate consent form. It is important to know that your participation in this study does not oblige you to take part in these other activities and interviews.

**Results of the study**

After we have completed the study and analyzed the information, we will inform you of the outcomes of the study through meeting(s) to be organized in Mwanza city. We will also inform local and national government of the findings. Depending on the outcome of the study BRAC may decide to continue to provide gender training session for other women in Mwanza.

**How will the information I give be kept private?**

All information collected in this study will be kept securely and confidential in a locked location. Your name and details will not be recorded on the notes written during the discussions. Analysis will only be done using the study number and your identity will remain private and confidential, unless we are required by law to release the information. Reports about the study and results will be presented to the organizations that are working together for this study and at scientific meetings. Results of this study may also be published in scientific journals. All presentations and publications of the findings of this study will not include any information which allows you to be identified as one of the study participants.

**What are the risks, stress or discomfort of taking part in this study?**

We do not expect that you will experience any harm by taking part in this study. However, some of the questions may be sensitive and you may feel embarrassed to discuss them with other people in the group. You can stop the interview or withdraw from the study if you feel uncomfortable.

If you decide you do not want to take part this will not affect this community being offered small financial loans or training on gender issues in the future. If you have any problems as a result of being in the study then you should discuss this with one of the persons named below.

**What are the benefits of participating?**

For those who are attending the training may benefit from the information about how to improve your relationship and your health more generally. For those who only attend the interview this study may have no direct benefit to you, however this research will help us to prepare training activities and materials that are acceptable and that may bring positive changes to the healthiness of relationships in this community. Therefore, your participation in this study will provide information which could help you and others in your community in the future. If you are subject to any violence during the course of the study, the study staff will provide information about available services within your community and refer you to the services if appropriate.

**Are there costs associated with participating in this study?**

There are no costs to you for participating in this study. For those of you who are in the group that will attend training, you will be given a reimbursement of Tanzanian shillings 2,500 after each training session. Everyone else who participates in the study will receive Tanzanian shillings 5,000 after each interview. This is compensation for your time, inconvenience and other costs which may be related to your participation in the study.

**Length of participation**

You will be asked to be interviewed around a week after signing this consent from. If you are selected to attend training this start around one month after you sign this consent from and will take place up to 6 months. The second interview will be conducted at some time later in 2015. It is therefore likely that your study participation will be approximately 24 months.

**Whom can I contact if I have questions or need additional information?**

We would like to answer all your questions. If you have any questions now, please ask us. If you have any questions later, you can also contact Dr Saidi Kapiga (Co-Investigator of this study and the Scientific Director of the Mwanza Intervention Trials Unit – MITU) or Dr Gerry Mshana (Co-Investigator of this study) at the following address:

Mwanza Intervention Trials Unit

National Institute for Medical Research

P.O. Box 11936

Mwanza, Tanzania

Telephone: 028-250 0019

If at any time you have any questions regarding your rights as a participant in this research study, you may contact Ms Joyce Ikingura at the address shown below:

Medical Research Coordinating Committee

National Institute for Medical research

P.O. Box 6953

Dar es Salaam, Tanzania

Telephone: 022-212 1400

**Agreement to join the study**

I have read this form, or had it read and explained to me. I understand the information and was able to ask all my questions. I have been given a copy of this form.

I voluntarily agree to participate in this study by signing below. If I am illiterate, I agree that a witness will underwrite and sign on my behalf.

Participant is illiterate Participant is literate

in Swahili in Swahili

Participant name (**print**) Participant signature/Thumbprint Date

Name of study staff conducting Study Staff signature Date

consent discussion (**print**)

Witness name* **(print)** Witness signature* Date*

(***Needed only if participant is illiterate**)

**Additional File 2**

**A cluster randomised controlled trial to assess the incremental impact on intimate partner violence of adding a 10-session participatory gender training programme to an existing microfinance intervention for women in Tanzania (MAISHA CRT01): study protocol**

**PARTICIPANT INFORMATION AND CONSENT FORM FOR MAISHA CRT01
IN-DEPTH INTERVIEW**

Version 1.1, 17 April 2015

**INTRODUCTION**

We are conducting research on an important issue related to health and healthy relationships. This research is being conducted by the National Institute for Medical Research (NIMR), Mwanza centre and the Mwanza Intervention Trials Unit (MITU) in collaboration with the London School of Hygiene & Tropical Medicine (LSHTM). We have been approved to conduct this research by the Tanzania Ministry of Health and Social Welfare and by the LSHTM ethics committee.

The purpose of the study is to explore ways to improve relationships and health more generally. In this study a number of women who are members of BRAC groups will be selected by chance to receive training on gender issues, using methods which allow active participation during the training sessions. Other women will be selected to form a group and by chance to receive training on gender issues, also using methods that allow active participation during the training sessions. For these women, separate training sessions on issues related to gender will be organized for their male partners, if these women agree to their partners being involved. In order to measure whether loans and training or training alone impact on improving healthy relationships and health, we will also recruit a number of women to form a group who will only be asked to attend discussions. These women will not receive either small financial loans or training on gender issues, during this study.

After two years of implementing these activities, we will assess the impact on relationships for the women in the study as well as other health related outcomes. We will also measure changes on the ability of women to have control over their everyday lives, and the economic and health benefits. We will also assess the cost of implementing these activities against their effectiveness.

**IN-DEPTH INTERVIEWS**

As part of the study we are planning to conduct in-depth interviews to help us gain deeper understanding of issues related to healthy relationships. This will involve a detailed conversation about health and healthy relationships.

We would like to hear your views and experiences about these issues. We would also like your views on other issues including the roles of women and men in financial issues, family life, as well as your views on violence against women. If you are part of the main study and have attended gender training or received microfinance we would also like to hear your views and experiences of these.

After you have had all your questions answered and feel you understand what you will have to do, you will be asked to sign, or put your thumb print on this consent form. The researcher will ask you for permission to record the interview. If you do not want the interview to be recorded then hand-written notes of the interview will be taken instead. The interview will be informal and you will be encouraged to talk freely about anything you feel is related to the questions about health and healthy relationships.There are no right and wrong answers and all your views will be respected.

The interview should take no longer than 2 hours, and will be conducted in a private place. If the interview takes longer and you need to leave to attend other duties, please feel free notify the interviewer and you may plan to continue with the interview some other time.

**DO I HAVE TO TAKE PART?**

This form provides information about the procedures. After reading and talking about the information provided with the researcher, you will be able to decide whether you want to take part in this study. If you decide to take part, we will ask you to sign this consent form.

Please note that:

- Your decision to take part is entirely voluntary. It is completely up to you to decide whether to take part in this interview.
- You may decide not to take part, and not lose any of your rights or benefits.
- If you decide to take part, you may drop out at any time, for any reason, without losing any rights or benefits.

**WHY HAVE I BEEN CHOSEN TO TAKE PART IN THE STUDY?**

You are invited to participate in an in-depth interview because you represent one of the groups who will be participating in the main study or because you are working in health or welfare services. We hope that you will be interested to participate and provide information relevant to the research.

**WHAT WILL YOU BE ASKED TO DO IF YOU DECIDE TO TAKE PART IN THE INTERVIEW?**

If you decide to participate in this interview, you may be asked to take part in other in-depth interviews in the future. You will be contacted and requested for consent to participate before these interviews. Your participation in these other interviews will be completely voluntary and you can decide not to participate in the future even if you agreed to participate today. You will be interviewed after signing the consent form. If you are invited to attend further interviews they will be conducted in about six month’s time and a year after that. It is therefore likely that your study participation will be for approximately 18 months.

**How will the information I give be kept private?**

Your contact details will be confidential and only be available to the staff involved in the study. The copy of the voice recording will not have your name attached to it. All information collected in this study will be kept securely and confidential in a locked location. Your name and details will not be recorded on the notes written during the interview or in reports from the study and your identity will remain private and confidential. Reports about this study may quote some of the words you tell us. Any words that you have told us during the interviews will not have your name attached to them.

**What are the risks, stress oR discomfort of taking part in this interview?**

We do not expect that you will experience any harm by taking part in this interview. However, some of the questions may be sensitive and you may feel embarrassed to discuss them. You can stop the interview or withdraw from this interview if you feel uncomfortable.

If you decide you do not want to take part this will not affect this community being offered small financial loans or training on gender issues in the future. If you have any problems as a result of being in the study then you should discuss this with one of the persons named below.

**WHAT ARE THE BENEFITS OF PARTICIPATING?**

We hope that the information gained from the interviews will improve our understanding of ways to improve healthy relationships in the community and reduce violence against women.

**Are there costs associated with participating in this study?**

There are no direct costs to you for participating in this study. You will receive Tanzanian Shillings 5,000 as a contribution towards the cost of your travel and time.

**HOW WILL I HEAR THE RESULTS OF THE STUDY?**

After we have completed the research we will inform you of the outcomes of this study together with the main study through meetings which will be organized in Mwanza city. We will also inform local and national government of the findings.

**Whom can I contact if I have questions or need additional information?**

We would like to answer all your questions. If you have any questions now, please ask us. If you have any questions later, you can also contact Dr Saidi Kapiga (Co-Investigator of this study and the Scientific Director of the Mwanza Intervention Trials Unit – MITU) or Dr Gerry Mshana (Co-Investigator of this study) at the following address:

Mwanza Intervention Trials Unit

National Institute for Medical Research

P.O. Box 11936

Mwanza, Tanzania

Telephone: 028-250 0019

If at any time you have any questions regarding your rights as a participant in this research study, you may contact Ms Joyce Ikingura at the address shown below:

Medical Research Coordinating Committee

National Institute for Medical research

P.O. Box 6953

Dar es Salaam, Tanzania

Telephone: 022-212 1400

### WHAT AM I REQUIRED TO DO?

If you agree to participate in this research, please sign below.

**Agreement to join the study**

I have read this form, or had it read and explained to me. I understand the information and was able to ask all my questions. I have been given a copy of this form.

I voluntarily agree to participate in this study by signing below. If I am illiterate, I agree that a witness will underwrite and sign on my behalf.

Participant is illiterate Participant is literate

in Swahili in Swahili

Participant name (**print**) Participant signature/Thumbprint Date

Name of study staff conducting Study Staff signature Date

consent discussion (**print**)

Witness name* **(print)** Witness signature* Date*

(***Needed only if participant is illiterate**)

**Additional File 3**

**A cluster randomised controlled trial to assess the incremental impact on intimate partner violence of adding a 10-session participatory gender training programme to an existing microfinance intervention for women in Tanzania (MAISHA CRT01): study protocol**

**PARTICIPANT INFORMATION AND CONSENT FORM FOR MAISHA CRT01**

**FOCUS GROUP DISCUSSION**

Version 1.1, 24 March 2015

**INTRODUCTION**

We are conducting research on an important issue related to health and healthy relationships. This research is being conducted by the National Institute for Medical Research (NIMR), Mwanza centre and the Mwanza Intervention Trials Unit (MITU) in collaboration with the London School of Hygiene & Tropical Medicine (LSHTM). We have been approved to conduct this research by the Tanzania Ministry of Health and Social Welfare and by the LSHTM ethics committee.

The purpose of the study is to explore ways to improve relationships and health more generally. In this study a number of BRAC groups will be selected by chance to receive training on gender issues using methods which allow active participation during the training sessions. Other women will be selected to form a group and by chance to receive training on gender issues also using methods that allow active participation during the training sessions. For these women, separate training sessions on issues related to gender issues will be organized for their male partners if these women agree to their partners being involved. In order to measure whether loans and training or training alone impact on improving healthy relationships and health; we will also recruit a number of women to form a group who will only be asked to attend discussions. These women will not receive either small financial loans or training on gender issues, during this study.

After one year of implementing these activities, we will assess the impact on relationships for the women in the study as well as other health related outcomes. We will also measure changes on the ability of women to have control over their everyday lives, and the economic and health benefits. We will also assess the cost of implementing these activities against their effectiveness.

**PARTICIPATORY GROUP DISCUSSIONS**

As part of the study we are planning to conduct participatory group discussions to help us gain more understanding of issues related to healthy relationships. This will involve detailed discussions and involvement with activities about health and healthy relationships in a group of individuals.

We would like to hear about your views and experiences about health and healthy relationships. We would also like your views on other issues including the roles of women and men in financial issues, family life, as well as your views on violence against women. If you are part of the main study and have attended gender training or received microfinance we would also like to hear your views and experiences of these.

Information from these participatory group discussions will help us to gain an in-depth understanding of health and healthy relationships and evaluate the effect of the project activities on improving these. If you are part of the main study and have attended gender training or received microfinance we would also like to understand the impact of these on your health and relationships.

After you have had all your questions answered and feel you understood what you will have to do, you will be asked to sign, or put your thumb print on this consent form. The researcher will ask you for permission to record the discussion and activities. If you do not want to be recorded but others in the group agree, you will be free to decline participating in the discussion. The discussions will be informal and you will be encouraged to talk freely about anything that you feel is related to the questions about health and healthy relationships.There are no right and wrong answers and all your views will be respected.

The discussion should take no longer than 2 hours and will be conducted in a private place suitable to you. If the discussion takes longer and you need to leave to attend other duties, please feel free to notify the research moderator or the note taker.

**DO I HAVE TO TAKE PART?**

This form provides information about the procedures. After reading and talking about the information provided with the researcher, you will be able to decide whether you want to take part in this study. If you decide to take part, we will ask you to sign this consent form.

Please note that:

- Your decision to take part is entirely voluntary. It is completely up to you to decide whether to take part in this participatory group discussion.
- You may decide not to take part, and not lose any of your rights or benefits.
- If you decide to take part, you may drop out at any time, for any reason, without losing any rights or benefits.

**WHY HAVE I BEEN CHOSEN TO TAKE PART IN THE STUDY?**

You are invited to participate in a participatory group discussion because you are participating in the main study or because you live in the neighbourhood area where the main study is being conducted. We hope that you will be interested to participate and provide information relevant to the research.

**WHAT WILL YOU BE ASKED TO DO IF YOU DECIDE TO TAKE PART IN THE PARTICIPATORY GROUP DISCUSSION?**

If you decide to participate in this participatory group discussion, you may be asked to take part in other discussions in the future. You will be contacted and requested for consent to participate before these participatory group discussions. Your participation in these other discussions will be completely voluntary and you can decide not to participate in the future even if you agreed to participate today. You will be asked to participate in the discussions after signing the consent form. If you are invited to attend further discussions they will be conducted in about six month’s time and a year after this discussion. It is therefore likely that your study participation will be for approximately 18 months.

**How will the information I give be kept private?**

Your contact details will be confidential and only be available to the staff involved in the study. The copy of the recording will not have your names attached to it. All information collected in this study will be kept securely and confidential in a locked location. Your names and details will not be recorded on the notes written during the discussions or in reports from the study and your identity will remain private and confidential. Reports about this study may quote some of the words you tell us. Any words that you have told us during the discussion will not have your name attached to them.

**What are the risks, stress oR discomfort of taking part in this discussion?**

We do not expect that you will experience any harm by taking part in this discussion. However, some of the questions may be sensitive and you may feel embarrassed to discuss them with others in a group. You are free not to answer any questions or to withdraw from this discussion if you feel uncomfortable.

If you decide you do not want to take part this will not affect this community being offered small financial loans or training on gender issues in the future. If you have any problems as a result of being in the study then you should discuss this with one of the persons named below.

**WHAT ARE THE BENEFITS OF PARTICIPATING?**

We hope that the information gained from the discussions will improve our understanding of ways to improve healthy relationships in the community and reduce violence against women.

**Are there costs associated with participating in this study?**

There are no costs to you for participating in this study. However, everyone who participates in this study will receive Tanzanian Shillings 5,000 as a contribution towards the cost of your travel and time.

**HOW WILL I HEAR THE RESULTS OF THE STUDY?**

After we have completed the research we will inform you of the outcomes of the main study through meetings which will be organized in Mwanza city. We will also inform local and national government of the findings.

**Whom can I contact if I have questions or need additional information?**

We would like to answer all your questions. If you have any questions now, please ask us. If you have any questions later, you can also contact Dr Saidi Kapiga (Co-Investigator of this study and the Scientific Director of the Mwanza Intervention Trials Unit (MITU) or Dr Gerry Mshana (Co-Investigator of this study) at the address:

Mwanza Intervention Trials Unit

National Institute for Medical Research

P.O. Box 11936

Mwanza, Tanzania

Telephone: 028-250 0019

If at any time you have any questions regarding your rights as a participant in this research study, you may contact Ms Joyce Ikingura at the address shown below:

Medical Research Coordinating Committee

National Institute for Medical research

P.O. Box 6953

Dar es Salaam, Tanzania

Telephone: 022-212 1400

### WHAT AM I REQUIRED TO DO?

If you agree to participate in this research, please sign below.

**Agreement to join the study**

I have read this form, or had it read and explained to me. I understand the information and was able to ask all my questions. I have been given a copy of this form.

I voluntarily agree to participate in this study by signing below. If I am illiterate, I agree that a witness will underwrite and sign on my behalf.

Participant is illiterate Participant is literate

in Swahili in Swahili

Participant name (**print**) Participant signature/Thumbprint Date

Name of study staff conducting Study Staff signature Date

consent discussion (**print**)

Witness name* **(print)** Witness signature* Date

(***Needed only if participant is illiterate**
