# Supplementary material for: Pathways to reduced physical intimate partner violence among women in north-western Tanzania: Evidence from two cluster randomised trials of the MAISHA intervention
Source: PLOS Glob Public Health. 2023 Nov 13;3(11):e0002497. doi: 10.1371/journal.pgph.0002497 (PMC10642778; doi:10.1371/journal.pgph.0002497)
Supplement: S2 Protocol — (DOCX) [file pgph.0002497.s008.docx]

**A cluster randomised controlled trial to assess the impact on intimate partner violence of a 10-session participatory social empowerment intervention for women in Tanzania (MAISHA CRT02): study protocol**

Sheila Harvey, BSc (Hons), MSc, PhD *
Department of Global Health and Development, London School of Hygiene & Tropical Medicine, 15-17 Tavistock Place, London WC1H 9SH and Mwanza Intervention Trials Unit, PO Box 11936, Mwanza, Tanzania
[sheila.harvey@lshtm.ac.uk](mailto:sheila.harvey@lshtm.ac.uk)

Shelley Lees, MSc, MRes, PhD
Department of Global Health and Development, London School of Hygiene & Tropical Medicine, 15-17 Tavistock Place, London WC1H 9SH [shelley.lees@lshtm.ac.uk](mailto:shelley.lees@lshtm.ac.uk)

Gerry Mshana, PhD
National Institute for Medical Research, PO Box 1462, Mwanza, Tanzania
[gerrymshana@hotmail.com](mailto:gerrymshana@hotmail.com)

Christian Hansen, BSc, MSc, PhD
Department of Infectious Disease Epidemiology, London School of Hygiene & Tropical Medicine, Keppel Street, London WC1E 7HT and Mwanza Intervention Trials Unit, PO Box 11936, Mwanza, Tanzania
[christian.hansen@lshtm.ac.uk](mailto:christian.hansen@lshtm.ac.uk)

Tanya Abramsky

Department of Global Health and Development, London School of Hygiene & Tropical Medicine, 15-17 Tavistock Place, London WC1H 9SH
[tanya.abramsky@lshtm.ac.uk](mailto:tanya.abramsky@lshtm.ac.uk)

Charlotte Watts, PhD, FMedSci
Department of Global Health and Development, London School of Hygiene & Tropical Medicine, 15-17 Tavistock Place, London WC1H 9SH [charlotte.watts@lshtm.ac.uk](mailto:charlotte.watts@lshtm.ac.uk)

Saidi Kapiga, MD, MPH, ScD
Mwanza Intervention Trials Unit, PO Box 11936, Mwanza, Tanzania and
Department of Infectious Disease Epidemiology, London School of Hygiene & Tropical Medicine, Keppel Street, London WC1E 7HT
[saidi.kapiga@lshtm.ac.uk](mailto:saidi.kapiga@lshtm.ac.uk)

*corresponding author

**Key words:** cluster randomized controlled trial, intimate partner violence, social empowerment

**Word count (main text):** 4,529

**Citation for this paper:** Harvey S, Lees S, Mshana G. A cluster randomised controlled trial to assess the impact on intimate partner violence of a 10-session participatory social empowerment intervention for women in Tanzania (MAISHA CRT02): study protocol, 2019. Available: http:// strive.lshtm.ac.uk/resources/cluster-randomised-controlled-trial-assess-impact-intimate-partner-violence-10-session

**ABSTRACT**

**Introduction:** Violence against women is a global public health problem. Worldwide, almost one third (30%) of women who have been in a relationship have experienced physical and/or sexual violence from an intimate partner. As well as being a human rights abuse, the negative short- and long-term impacts on women’s physical and mental health are considerable. There is an urgent need for rigorous evidence on violence prevention interventions.

**Methods and analysis:** The study, conducted in Mwanza city, Tanzania, comprises a cluster randomised controlled trial (RCT), a longitudinal qualitative study and integrated process evaluation. It is assessing the impact of a participatory social empowerment intervention on women’s experience of intimate partner violence (IPV). Sixty-six neighbourhood groups of women are being formed and randomly allocated to: participate in the 10-session MAISHA intervention (n=33); or, to be wait-listed for the intervention post-trial (n=33). Study participants are interviewed at baseline and 24 months post-intervention about their: household; partner; income; health; attitudes and social norms; relationship; childhood; and community. For the qualitative study and process evaluation, focus group discussions are being conducted with study participants and MAISHA intervention facilitators, and in-depth interviews with a purposive sample of 18 participants. The primary outcomes, assessed at 24 months post-intervention, are women’s reported experience of past year physical IPV and sexual IPV. Secondary outcomes include: reported experience of past year emotional abuse, acceptability and tolerance of IPV, and reported disclosure of IPV to others.

**Ethics and dissemination:** The study protocol has been approved by the Ethics Committees of the Tanzanian National Institute for Medical Research (Ref: NIMR/HQ/R.8a/Vol. IX/152) and London School of Hygiene & Tropical Medicine (Project ID: 11642). The study is being conducted following World Health Organisation recommendations on researching violence against women.

**Trial registration:** Registry for International Development Impact Evaluation (RIDIE-STUDY-ID-52546f0039f52) and ClinicalTrials.gov ID: NCT02592252.

**INTRODUCTION**

**Background and rationale**

Violence against women has been described by the World Health Organisation (WHO) as a “global public health problem of epidemic proportions” (1). The most common form of violence against women is intimate partner violence (IPV) with estimates from population surveys indicating that, worldwide, around a third of women will experience physical and/or sexual violence from an intimate partner in their lifetime (2). The past decade has seen a rapidly growing body of research highlighting multiple negative impacts of IPV, not only on the physical and mental health of the women affected (1, 3-7), but that of their children (8), families and communities. Furthermore, there is mounting evidence of an association between violence in all its forms (physical, sexual, and emotional) and increased risk of HIV infection among women (9, 10).

Although the specialty of violence prevention is still at a relatively early stage, evidence on effective violence prevention interventions is emerging. In a synthesis of existing reviews of evidence on violence prevention interventions, Ellsberg et al (2015) found that data are highly skewed towards studies conducted in high-income countries with intervention research focused more on response than prevention. However, they also highlighted a growing body of rigorous evidence from sub-Saharan Africa showing that IPV is preventable (11). One of the first randomised controlled trials (RCT) from this region was the Intervention with Microfinance for AIDS & Gender Equity (IMAGE) trial, implemented in rural South Africa (12). The intervention combines group-based microfinance with a participatory gender and HIV training programme. In a cluster RCT IMAGE was shown, over a two-year period, to reduce women’s past year experience of physical and/or sexual IPV by 55% (12). In addition, levels of household poverty were significantly reduced and participants were more empowered as evidenced by greater self-confidence, autonomy in decision making, and increased ability to challenge gender norms when compared with women in the control population (13, 14).

Regional and international policy makers have asked whether, with appropriate national level refinement and adaptation, the IMAGE model would achieve the same level of impact if it was implemented in other sub-Saharan African settings. Because the IMAGE model is a combined microfinance-training intervention, the trial results also raise other policy- and programme-related questions, including, the extent to which the observed effect on IPV is attributable to the different components of the IMAGE model. To address this question, Kim et al (2009) analysed cross-sectional data from villages in the IMAGE trial that received the combined intervention (IMAGE), matched villages receiving microfinance alone, and a control group of villages receiving no intervention. Only the combined (IMAGE) intervention (microfinance and training) was associated with consistent reductions in different indicators of violence compared with microfinance only, and with no intervention. When microfinance only was compared with no intervention the direction of intervention effects varied across the different indicators of violence suggesting that the participatory gender training component of the IMAGE intervention is the key component in broadening the health and social effects of economic interventions such as microfinance (13).

The MAISHA study seeks to respond to the questions raised by the IMAGE trial. Population surveys indicate high levels of ongoing IPV against women in Tanzania (15, 16). The WHO multi-country study on women’s health and domestic violence found that almost 30% of ever-partnered women in a rural area of Tanzania had experienced physical and/or sexual violence IPV in the year prior to the survey (15). MAISHA is a mixed methods study that includes two cluster RCTs. The first RCT (MAISHA CRT01) is described in a separate paper (17) and seeks to assess whether an adapted 10-session social empowerment intervention can be integrated into an existing microfinance programme and have an impact on women’s experience of IPV. The second RCT (MAISHA CRT02) is described in this paper and seeks to assess the impact of the same 10-session social empowerment intervention on IPV among women not engaged in a microfinance programme. Data from the two RCTs will address the questions raised by IMAGE (12). First, by assessing whether a similar intervention with appropriate adaptation can have a similar impact in another setting, and second, to understand better the relative and combined effects of economic empowerment and social empowerment interventions on women’s experience of IPV.

In this paper, we describe MAISHA CRT02 which is evaluating the impact on IPV of a social empowerment intervention for women not engaged in a formal microfinance scheme. The study is being implemented by the Tanzanian National Institute for Medical Research (NIMR), Mwanza Intervention Trials Unit (MITU) and London School of Hygiene & Tropical Medicine (LSHTM).

**Aim and objectives**

The overall aim of the study is to assess the impact of a participatory social empowerment intervention delivered to newly-formed neighbourhood groups of women on their past year experience of IPV. The main objectives are to assess the impact of the intervention on past year physical IPV, sexual IPV, emotional abuse, women’s attitudes and beliefs around IPV, and on women’s past year disclosure of violence to others (by women who report experiencing past year physical and/or sexual IPV). Additional objectives, to be addressed through a complementary longitudinal qualitative study, are to learn more about the factors that contribute to women’s vulnerability to violence, and to understand how the intervention impacts on the lives of the participants and their families.

The theory of change model (Fig 1) maps out the key contextual factors that may influence the impact of the intervention, the components of the intervention, the expected initial, intermediate and longer-term outcomes of the intervention, and the overall impact the intervention is designed to have on women in Tanzania.

Figure 1 Theory of Change


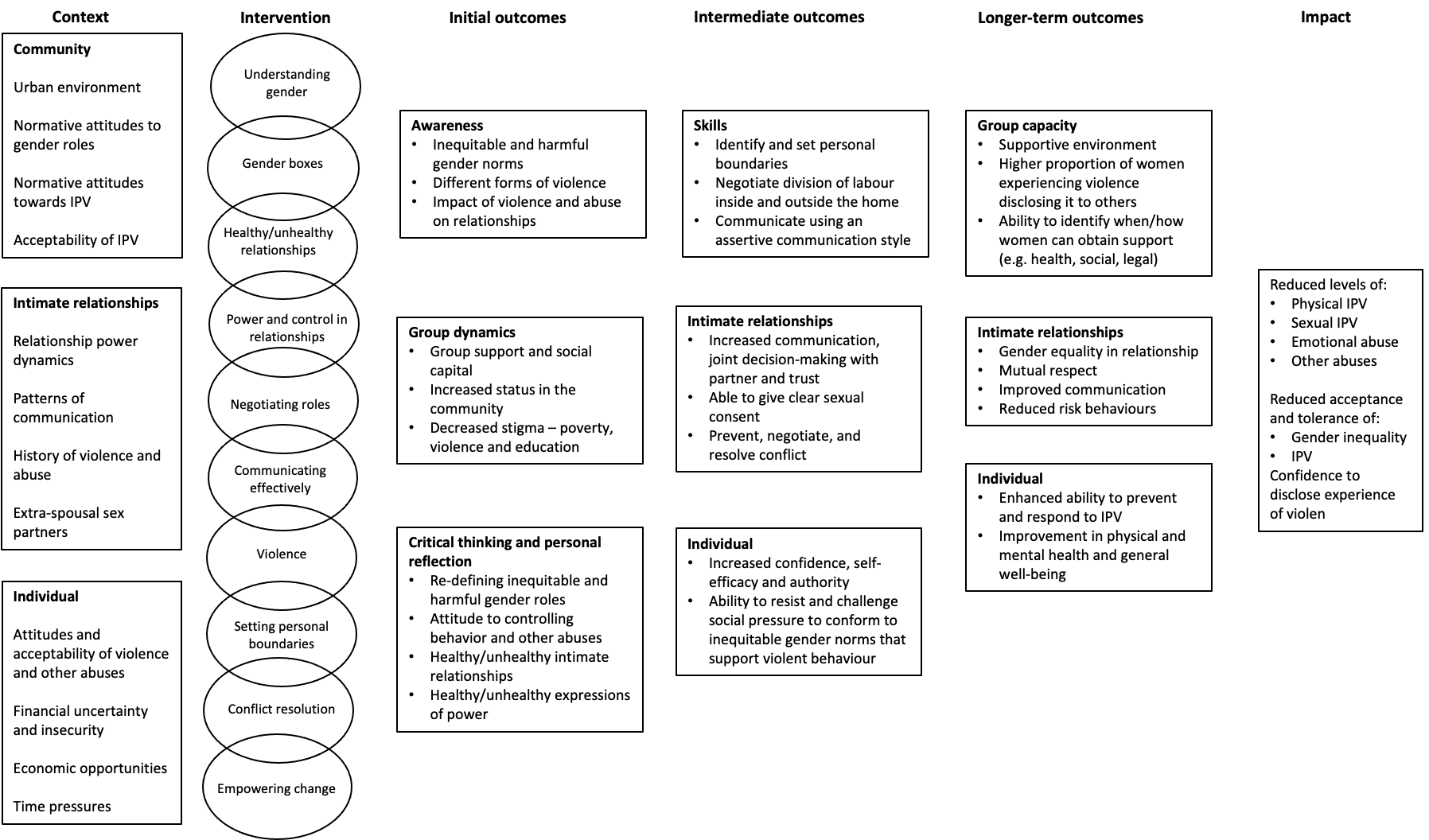


**METHODS AND ANALYSIS**

**Study design and setting**

This is a mixed methods study comprising a cluster RCT with a complementary longitudinal qualitative study and integrated process evaluation. The study is being conducted in Mwanza city in northwestern Tanzania.

**Participant and public involvement**

The design of the MAISHA study was inspired by IMAGE and aims to address questions raised by the trial in South Africa. Although there was not specific public involvement in development of the research question and outcomes, discussions were held with local community leaders and representatives about the purpose of the study. The trial team works in collaboration with local community leaders to identify neighbourhoods across the city in which to form women’s groups. Within a neighbourhood, the study team work closely with local leaders to identify and invite women to local community information meetings about the study. Women who are interested in taking part are then invited to further meetings to receive more detailed information about the study, to be assessed for eligibility, and to undergo informed consent procedures. The process of randomisation (described in more detail below) is a participatory process that involves participant representatives to ensure transparency in allocation of groups of women to each arm of the trial. Community advisory committees have been established that comprise local leaders, religious leaders, trial participants, and other relevant stakeholders. These meet regularly to facilitate effective communication between the trial team and study communities, and to ensure that any concerns from members of the community are addresses promptly. Results of the trial will be disseminated first to the trial participants and then to community advisory committees and local stakeholders before wider dissemination to national and international stakeholders.

**Eligibility criteria**

Women who meet the following criteria are eligible for inclusion in the study:

1. aged 20-50 years;
2. not formally employed, i.e. self-employed or not currently working;
3. resident in Mwanza for at least two years;
4. not been a member of formal microfinance loan group scheme in the past 12 months;
5. fluent in Swahili; and
6. have consented to participate in the study, that is they:
7. have demonstrated comprehension of the study procedures;
8. are willing to undergo the study procedures, including participating in the 10 intervention sessions, if randomly assigned to this arm of the trial; and
9. have signed an informed consent form.

Eligible women who provide informed consent are invited to join a neighbourhood group of around 15 to 20 women.

**Intervention and comparator**

The MAISHA intervention is a 10-session participatory social empowerment intervention delivered to women in groups. Groups of women in the comparator arm are wait-listed for the intervention following completion of study activities. The intervention follows the *Wanawake na Maisha* (which means “women and life” in Kiswahili) curriculum, which was developed by EngenderHealth in collaboration with the study team drawing on other published curricula (12, 18-22), including the *Sisters for Life* curriculum (12) which forms part of IMAGE. The MAISHA intervention is designed to be participatory and reflective, and aims to empower women, prevent IPV, and promote healthy relationships by: increasing knowledge and awareness (e.g. of the consequences of normative attitudes to gender and IPV); developing relationship skills (e.g. communication and conflict resolution); and creating group dynamics and stability (e.g. increased peer support and social capital). The intended outcomes for participants completing the intervention are detailed in Table 1.

Table 1 Intended outcomes for participants completing the MAISHA intervention

| **Intended outcomes for participants is that on completion of *Wanawake Na Maisha*, they should be able to:**   - Identify inequitable and harmful gender norms that exist in their community, especially those norms that contribute to intimate partner violence - Explain how abiding to inequitable and harmful gender norms has health and social costs to women, men, families and the community - Re-define inequitable and harmful gender norms into equitable and healthy alternatives - Describe the characteristics of healthy and unhealthy romantic relationships - Explain why controlling and abusive behaviour is unhealthy in a romantic relationship - Explain healthy and unhealthy expressions of power - Identify, set and manage personal boundaries - Negotiate division of labour inside and outside the home - Communicate using an assertive communication style - Identify different forms of violence including emotional, physical, economic and sexual violence - Explain the impact of intimate partner violence on the health and wellbeing of women, men, families and communities - Give clear sexual consent - Prevent, negotiate and resolve conflict - Resist and challenge social pressure to conform to inequitable gender norms that support violent behaviour - Identify when and how women can obtain support against violence (e.g. health, social, legal, etc.), if needed |
| --- |

The MAISHA curriculum is delivered on alternate weeks over a 20-week period. Each of the 10 sessions (outlined in Fig 1) is approximately an hour and a half to two hours giving a total time of approximately 15-20 hours. Each session is participatory and comprises: giving information to participants, small group activities, and group discussions, ending with a take home assignment designed to encourage participants to practice the skills covered during the session. Sessions are facilitated by trained facilitators following the *Wanawake na Maisha* curriculum manual, which provides detailed guidance for each session. The manual includes tips and notes for the facilitators, including examples of group ice-breakers and energisers. The facilitators have been trained by EngenderHealth with the training programme encompassing:

- gender equitable behavior and attitudes;
- discussions around beliefs, including the belief that intimate relationships should never be coercive, exploitative or abusive, belief in the importance of gender equity and women’s rights, and belief that inequitable gender norms can be changed;
- managing group dynamics, including emotional reactions and disclosure of sensitive information;
- establishing a safe and comfortable learning environment;
- encouraging all participants to take part in discussions.

Ongoing training of the MAISHA intervention facilitators, including practicing facilitation skills through role play, is supported by MITU and LSHTM. The facilitators are not involved in collection of baseline data, or any study outcome assessments.

**Outcomes**

The primary outcomes are women’s reported past year physical IPV and sexual IPV assessed 24 months after completion of intervention activities. Other outcomes to be examined are past year emotional abuse by a partner, IPV related attitudes and beliefs (attitudes condoning men’s use of violence against their female partners, the belief that a woman should tolerate violence in order to keep her family together, and the belief that IPV is a private matter in which others should not intervene), and past year disclosure of IPV (by women who report past year experience of physical/sexual IPV). Details of questionnaire items used to construct the outcomes are presented in Table 2. These were adapted from the WHO Violence Against Women instrument (15).

Table 2 Questions used to construct primary and secondary outcomes

| **Outcome** | **Questions** |
| --- | --- |
| Physical IPV | Reported that her current or any other partner has done at least one of the following things to her in the past 12 months:   - Slapped her or thrown something at her that could hurt her - Pushed her or shoved her or pulled her hair - Hit her with his fist or something else that could hurt her - Kicked her, dragged her or beat her up - Choked or burnt her on purpose - Threatened to use or actually used a gun, knife or other weapon against her |
| Sexual IPV | Reported that at least one of the following things has happened to her in the past 12 months:   - Current or any other partner forced her to have sexual intercourse by threatening her, holding her down or hurting her in some way - She had sexual intercourse when she did not want to because she was afraid that her partner would hurt her or someone she cared about if she refused - She had sexual intercourse when she did not want to because she was afraid that her partner would leave her or take another girlfriend if she refused |
| Emotional abuse | Reported that her current or any other partner has done at least one of the following things to her in the past 12 months:   - Insulted her or made her feel bad about herself - Belittled or humiliated her in front of other people - Done things to scare or humiliate her on purpose (e.g. by the way he looked at her, by yelling and smashing things) - Verbally threatened to hurt her or someone she cares about |
| Disclosure of IPV (among women who physical and/or sexual IPV in the past 12 months) | Reported that she has told someone within the past 12 months about her partner’s behaviour (violence/abuse) towards her |
| Attitudes accepting of IPV | Reported that she ‘strongly agrees’ or ‘agrees’ that a man has good reason to hit his wife in at least one of the following scenarios:   - She does not complete her household work to his satisfaction - She disobeys him - She refuses to have sexual intercourse with him - She protests because he has other girlfriends - He suspects that she is unfaithful in marriage - He finds out that she has been unfaithful in marriage |
| Believes a woman should tolerate violence in order to keep her family together | Reported that she ‘strongly agrees’ or ‘agrees’ with the statement:  ‘A woman should tolerate violence in order to keep her family together’ |
| Believes IPV is a private matter | Reported that she ‘strongly agrees’ or ‘agrees’ with the statement:  ‘Violence between husband and wife is a private matter and others should not intervene.’ |

**Participant timeline**

Following enrolment into the trial, baseline data are collected from women who have consented to take part. Randomisation occurs once six groups have been formed and all women in the six groups have completed the baseline interview. The intervention is delivered over 20 weeks (five months) and women in both arms are then followed up 24 months later, after completion of intervention activities, i.e. 29 months post-randomisation (Fig 2).

Figure 2 Participant Flow Through the Trial


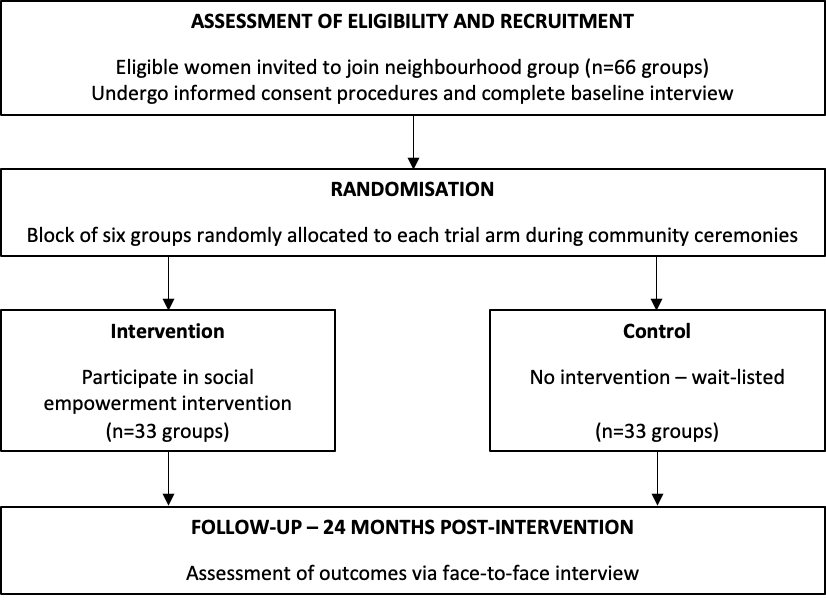


**Sample size**

The original sample size calculation was based on a composite outcome of past year physical and/or sexual IPV. It was estimated that a sample size of 33 groups per trial arm with an average of 20 participants per group (allowing for 10% loss to follow-up) would provide 80% power to detect a reduction of 30% past year physical and/or sexual IPV, and 90% power to detect a reduction of 34%, assuming an intra-cluster correlation of 0.02. This assumed an estimated prevalence of past year physical and/or sexual IPV of 30% in the comparison arm, based on data from the WHO multi-country study in Tanzania (15). In light of new evidence (see Protocol amendments below), it was decided by the trial management committee that the composite measure of physical and/or sexual IPV should be dropped as an outcome, and instead, to focus on the impact of the intervention on physical IPV and sexual IPV as separate outcomes. Among the 1248 women who completed the baseline interview, 25% (95% confidence interval 22% to 28%) reported past year physical IPV (unpublished data). A sample size of 33 groups per trial arm with an average of 20 participants per group (allowing for 10% loss to follow-up) will provide 80% power to detect a reduction of 33% in past year physical IPV, and 90% power to detect a reduction of 38%, assuming an intra-cluster correlation of 0.035. Even with an intra-cluster correlation of 0.05, the trial will have 80% power to detect a reduction in past year physical IPV of 35%.

**Recruitment and formation of women’s groups**

The study team is working in close collaboration with community leaders in neighbourhoods across Mwanza city. Once a neighbourhood has been identified for possible inclusion, MAISHA study team members hold meetings with local political, religious and community leaders to provide information about the overall aim and objectives of the study, eligibility to take part, and the study procedures, including enrolment procedures for women, delivery of the intervention, and assessment of outcomes. If the leaders agree to the study team working in the neighbourhood further meetings are held to provide information to the wider community. The study team work with the local leaders to identify and visit households in order to invite potentially eligible women to attend information meetings about the study. Women who are interested in taking part in the study are then invited to further meetings to receive more detailed information about the study, to be assessed for eligibility, and to undergo informed consent procedures.

**Informed consent**

Once a woman has been identified as meeting eligibility, the study team provides her with a copy of the participant information sheet and goes through it in detail allowing her time to ask questions about the study. If the woman agrees to participate and has demonstrated that she understands the study procedures, she is invited to sign the consent form. Participants who are invited to take part in the longitudinal qualitative study are given an additional participant information sheet. A member of the study team meets with the participant to go through the participant information sheet in detail, and to allow the participant time to answer ask any questions. If the participant agrees to participate in the qualitative study she is invited to sign an additional consent form.

**Allocation method and blinding**

The newly formed groups of women are randomised in blocks of six groups during community ceremonies. It is a two-stage process that is both participatory and transparent involving the study team and a representative from each group to be randomised. First, representatives from each of the six groups are randomly divided equally into two sets (A and B). This is done by each representative drawing a folded sheet of paper (with A or B written on it) from a box. Second, one representative is asked to call (heads or tails) for her set of three groups to be allocated to immediate intervention. A study team member then tosses a coin to randomly allocate each set of three groups to either immediate intervention (intervention arm) or to be wait-listed for the intervention post-study (control arm). Given the nature of the intervention, it is not possible, after assignment, to mask participants or the study team involved in day-to-day operations and delivery of the intervention. Data analysts are blinded to allocation.

**Data collection methods – quantitative**

The MAISHA study schedule is outlined in Table 3 (adapted from the SPIRIT template (23)). Data are collected at the following time points:

1. Baseline (prior to randomisation) – a face-to-face interview is conducted using a structured questionnaire adapted from the WHO Violence Against Women instrument (15). The questionnaire has seven sections which ask the woman about her household; partner; income; health; attitudes and social norms; relationship (including experiences of violence), childhood; and her community. The questionnaire has been translated into Kiswahili (the national language) and interviews are conducted in private by female interviewers trained in interviewing techniques, gender issues, violence, and ethical issues related to research on IPV (24, 25).

2. Intervention period – during the 20-week intervention period, the intervention sessions that each woman (in the intervention arm) participates in are recorded and where possible, reasons for non-participation are documented.

3. 29 months post-randomisation – a face-to-face interview conducted by trained female interviewers, using a structured questionnaire to assess outcomes, similar to that used at baseline and following the same procedures.

Table 3 MAISHA Study Schedule (based on SPIRIT template [23])

|  | **STUDY PERIOD** | | | | | | | |
| --- | --- | --- | --- | --- | --- | --- | --- | --- |
|  | **Enrolment** | **Allocation** | **Intervention** | | | | | **Closeout** |
| Time point (months) | ***- M1*** | ***0*** | ***M1*** | ***M2*** | ***M3*** | ***M4*** | ***M5*** | ***M29*** |
| **Enrolment** |  |  |  |  |  |  |  |  |
| Eligibility screen | x |  |  |  |  |  |  |  |
| Informed consent | x |  |  |  |  |  |  |  |
| **Allocation** |  | x |  |  |  |  |  |  |
| **Interventions** |  |  |  |  |  |  |  |  |
| No intervention (control) |  |  | x | x | x | x | x |  |
| PGT (intervention) |  |  | x | x | x | x | x |  |
| **Assessments** |  |  |  |  |  |  |  |  |
| ***Baseline:*** |  |  |  |  |  |  |  |  |
| Socio-demographics | x |  |  |  |  |  |  |  |
| Physical IPV ^a^ | x |  |  |  |  |  |  |  |
| Sexual IPV ^a^ | x |  |  |  |  |  |  |  |
| Emotional abuse ^a^ | x |  |  |  |  |  |  |  |
| Attitudes about IPV | x |  |  |  |  |  |  |  |
| Disclosure of IPV to others ^a^ | x |  |  |  |  |  |  |  |
| In-depth interview ^b^ |  | x |  |  |  |  |  |  |
| Focus group discussion ^b^ |  | x |  |  |  |  |  |  |
| ***Post-intervention:*** ^c^ |  |  |  |  |  |  |  |  |
| In-depth interview ^b^ |  |  |  |  |  |  | x |  |
| Focus group discussion ^b^ |  |  |  |  |  |  | x |  |
| ***Follow-up:*** |  |  |  |  |  |  |  |  |
| Socio-demographics |  |  |  |  |  |  |  | x |
| Physical IPV ^a^ |  |  |  |  |  |  |  | x |
| Sexual IPV ^a^ |  |  |  |  |  |  |  | x |
| Emotional abuse ^a^ |  |  |  |  |  |  |  | x |
| Attitudes about IPV |  |  |  |  |  |  |  | x |
| Disclosure of IPV to others ^a^ |  |  |  |  |  |  |  | x |
| In-depth interview ^b^ |  |  |  |  |  |  |  | x |
| Focus group discussion ^b^ |  |  |  |  |  |  |  | x |

PGT-participatory gender training; IPV-intimate partner violence;

^a^ Reported experience in past 12 months

^b^ Participants will be random sample of women from control and intervention arms – same women will participate at three time-points

^c^ immediately following completion of the MAISHA intervention activities

**Data collection methods – qualitative**

A total of 54 in-depth interviews (IDIs) are being conducted with participants. Eighteen women are purposefully selected from the two trial arms (12 intervention, 6 control) to represent women who do and do not report IPV at baseline. A separate team of trained interviewers conduct the IDIs and are blinded as to whether, or not, a woman has reported IPV. Each woman is invited to attend three IDIs – pre-intervention, immediately post-intervention and 24 months post-intervention. The IDIs explore the participants’ life stories, the socio-cultural and structural factors associated with IPV and personal experiences of IPV and its impact on both the women themselves and their children. For women in the intervention arm, the post-intervention IDIs also explore their views and experiences of the MAISHA intervention and its impact on their experiences of IPV. Five trial participants from the intervention arm who drop out of the intervention after participating in two sessions will be invited to participate in an IDI to explore their reasons for withdrawal from the MAISHA intervention.

Twenty-seven focus group discussions (FGDs) are being conducted – comprising nine FGDs at three time points (pre-intervention, immediately post-intervention and 24 months post-intervention). Six FGDs are being conducted with intervention arm groups and three with control arm groups. Where possible the same groups of women are asked to attend at all three time points. The FGDs explore experiences of the socio-cultural and structural factors associated with IPV. The post-intervention FGDs with women in the intervention arm also explore their views and experiences of the MAISHA intervention and its impact on their views of IPV.

Participatory observations are being conducted at selected MAISHA intervention sessions, ensuring that each session is observed at least once. Social scientists conduct informal conversations with study participants to assess their impression of the sessions and its immediate impact.

**Data management**

Questionnaire data collected from trial participants at baseline and at 24 months post-intervention are recorded directly onto a tablet computer. The electronic questionnaire form has in-built checks to minimise the level of missing data and to minimise entry of erroneous data. The data recorded on the tablet computer are uploaded to the study database daily and checked for missing and/or erroneous data. Any data queries are sent to the team leader to be resolved with the research assistants conducting the interviews.

Participation in the MAISHA intervention sessions and reasons for non-participation are recorded on paper and entered into the study database following double-entry data procedures. Data are checked for missing or erroneous data. Any data queries are sent to the team leader to be resolved with the MAISHA intervention facilitators.

All IDIs and FGDs with participants are audio recorded with the participant’s consent. Hand written notes are taken during the participatory observations of the MAISHA curriculum sessions. Audio recordings and hand written notes are transcribed and translated from Kiswahili (the national language) into English. A sample of the transcripts are checked for quality of transcription and translation. Transcripts are imported to the qualitative analysis package NVIVO (QSR International Pty Ltd, Doncaster, Australia).

All study data are stored in secure databases with restricted access. Each participant is allocated a unique study identifier. Names and other participant identifiers are not recorded in the study database. Paper records – e.g. consent forms, tracking forms with participant names and contact details – are stored securely in locked filing cabinets in secure offices within the study coordinating centre at MITU, which has 24-hour security and restricted access.

**Confidentiality**

Participants’ names and any information that could identify them is kept confidential. Women are allocated a unique study number. The questionnaires for the quantitative baseline and follow-up interviews are anonymous and responses to questions are entered directly onto a tablet computer. On the same day as the interview, data are uploaded to the secure study database and removed from the tablet computer before the next interview is conducted. Qualitative IDIs are audio recorded with the participant’s consent. The recordings are labelled with the study number only and are destroyed once the recording has been transcribed and translated to English. All identifiers will be destroyed at the end of the study.

**Statistical methods**

A detailed statistical analysis plan will be prepared prior to follow-up interviews. Data from the baseline interviews will be used to verify the sample size calculations and to identify differences between clusters. The coefficient of variation across clusters will be calculated based on the reported prevalence of IPV. Data from the baseline will also be used to identify important predictors for IPV and important health-related outcomes, such as poor mental health.

The primary trial analysis will adopt an intention to treat approach, assessing the impact of the intervention on women in the intervention arm 24 months after intervention activities have finished, irrespective of whether or not they received the full “dose” (i.e. participated in all 10 sessions) of the MAISHA intervention. Secondary analyses will be conducted to investigate differences in impact according to the “dose” of intervention received.

The primary outcome variables (self-reported experience of past year physical IPV and sexual IPV) will be analysed in a random intercepts logistic regression model to account for the clustered study design, and adjusted for differences in baseline characteristics where relevant. The same analysis will be conducted to examine the secondary outcome variables – self-reported experience of past year emotional abuse, IPV related attitudes and beliefs, and past year disclosure of violence to others (by women who report past year experience of physical and/or sexual IPV).

Steps have been taken to minimise contamination of the control arm which includes documenting the women who participate in the intervention sessions. The potential for direct and indirect contamination of control arm women will be investigated by asking women during follow-up if they participated in any of the MAISHA intervention sessions, or if they have discussed any of the sessions with other women participating in the MAISHA study.

**Safety monitoring**

Given that no outcome data (i.e. experiences of IPV) are collected during the five-month intervention period or during the period up to 24 months post-intervention, a data monitoring committee has not been established as no interim analyses are planned. The MAISHA study is being conducted following the WHO’s guidelines on researching violence against women (24). These guidelines are in place to minimise the risk of potential harm to women participating in the study. Female interviewers for the quantitative baseline and follow-up interviews, and for the qualitative IDIs and FGDs, have received training in interviewing techniques, gender issues, violence, and ethical issues related to research on IPV (24, 25). All participants are provided with information about organisations offering support to women (and their children, if appropriate) experiencing violence and other forms of abuse. Participants who report violence and other forms of abuse during the study are offered counseling by a trained member of the study team and referral to an appropriate organisation for ongoing support.

**Auditing**

Regular audits of the conduct of the study are carried out by members of the study team. These include checks that participant informed consent procedures have been followed correctly, observation and assessments of facilitation of the MAISHA intervention sessions, monitoring of women’s participation in the MAISHA intervention sessions, and follow-up of non-participants.

**Protocol amendments**

There have been three amendments to the protocol as follows:

1) The follow-up period was extended from 12 months post-intervention to 24 months post-intervention following confirmation of the additional funding required. This is considered to be a more appropriate time point at which to assess the effectiveness of the MAISHA intervention in reducing women’s past year experience of IPV. In addition, the secondary outcomes for the main trial report were reviewed and amended to ensure that they were clearly defined, specific and measurable.

2) The original design of MAISHA CRT02 was to invite the male partners of women in the groups allocated to the intervention arm to participate in a similar intervention designed for men. In light of the low numbers of male partners who agreed to participate, along with poor participation at intervention sessions among those who did consent to take part, and taking account of project resources, a decision was made by the trial management committee to terminate recruitment of male partners but to continue forming and enrolling groups of women. Work is continuing with men through a qualitative sub-study to learn about the facilitators and barriers for men’s participation in research and in participatory gender awareness programmes. The findings will be used to inform future research.

3) The primary outcome of the trial was changed from a composite of physical and/or sexual IPV to physical IPV and sexual IPV as separate primary outcomes. Evidence from other trials of violence prevention interventions (26, 27) have demonstrated limited impact on sexual IPV, a pattern that was hypothesised from the outset of the SASA! cluster RCT (26). Added to this, we recently found in the MAISHA CRT01 trial that while the MAISHA intervention led to marked reductions in past year physical IPV, the effect on past year sexual IPV was limited among women engaged in a formal group-based microfinance scheme (paper submitted). In light of this evidence, the trial management committee agreed that the composite measure of physical and/or sexual IPV should be dropped as an outcome. The remaining outcomes will be retained with physical IPV and sexual IPV as the primary outcomes of interest.

**Study status**

Sixty-six groups of women have been formed and delivery of the intervention is complete. Follow-up of women is ongoing and will continue through to the third quarter of 2019. The results of the study will be disseminated from late 2019 onwards.

The study described in this paper forms part of the MAISHA study, a programme of research that also includes: a second complementary cluster RCT (MAISHA CRT01) to evaluate the impact of the MAISHA intervention delivered to women engaged in formal group-based microfinance (17); an economic evaluation to evaluate the total costs of the development and implementation of the MAISHA intervention; and cross-sectional surveys of the male partners of women taking part in both RCTs to identify risk factors in men associated with IPV perpetration (e.g. alcohol use, employment, and abuse during childhood), and to explore whether the intervention delivered to women has impacted on their male partners’ attitudes and behavior.

**Ethics and dissemination**

The MAISHA study has been approved by the Ethics Committees of the Tanzanian National Institute for Medical Research (Ref: NIMR/HQ/R.8a/Vol. IX/152) and the London School of Hygiene & Tropical Medicine (Project ID: 11642). The study is being conducted following WHO recommendations on researching violence against women (24).

The study findings will be widely disseminated through both formal and informal mechanisms. Meetings will be held with the participants to inform them of the results of the study. For women in the control arm, information will be provided as to how the MAISHA intervention will be expanded into their communities, if it is shown to work. The study findings will be presented to key stakeholders at local, regional and national level in Tanzania and at relevant regional, national and international conferences and meetings. Reports of the study will be prepared by the study team for submission to peer-review scientific journals. Other strategies to facilitate dissemination of the results of the study will be developed through collaboration with organisations, consortia and forums such as STRIVE (Tackling the structural drivers of HIV) and the Sexual Violence Research Initiative (SVRI).

**Data**

The MAISHA dataset is currently unavailable as the study is ongoing.

**Author contributions**

CW, SK and SL designed the study and led the grant application. SH and SK provide methodological input and oversee the conduct of the study and management of the research teams. GM and SL oversee the longitudinal qualitative study. CH and TA provide statistical and methodological input. All authors contributed to the manuscript preparation, and approved the final manuscript for submission.

**ACKNOWLEDGEMENTS**

First and foremost, we wish to thank all study participants for their time and commitment to the study. We are also grateful to the MAISHA study team for their contribution and tireless dedication implementing the study in Tanzania, and to the administration teams at MITU and LSHTM for their support.

**Sponsor and funding**

Trial sponsor: London School of Hygiene & Tropical Medicine (Reference: QA430)

This work is supported by the STRIVE Research Programme Consortium funded by UK Aid from the Department for International Development (DFID), and another donor who wishes to remain anonymous. The views expressed in this paper do not necessarily reflect the department’s official policies. The funding bodies and sponsor have had no role in the design of the study, or in writing this manuscript, and will not have any role in its conduct, analysis and interpretation of the data, or decisions to disseminate the results.

**Trial Management Committee**

Study investigators; study coordinator; team leaders (baseline and follow-up survey team, intervention team), senior social scientist, senior data manager, senior community liaison officer.

Roles and responsibilities include: study planning; budget management; preparation of progress reports for the ethics committees and funder; recruitment; randomisation; delivery of intervention; data collection and verification; analysis of data.

**Competing interests**

Following initiation of the study, Professor Watts has been seconded to DFID as their chief scientific advisor. Her ongoing role in this study is in her academic capacity at LSHTM. No other interests declared.

**REFERENCES**

1. World Health Organization, London School of Hygiene & Tropical Medicine, South African Medical Research Council. Global and regional estimates of violence against women: prevalence and health effects of intimate partner violence and non-partner sexual violence. Geneva; 2013.

2. Devries KM, Mak JY, Garcia-Moreno C, Petzold M, Child JC, Falder G, et al. Global health. The global prevalence of intimate partner violence against women. Science. 2013;340(6140):1527-8.

3. Ellsberg M, Jansen HA, Heise L, Watts CH, Garcia-Moreno C, Health WHOM-cSoWs, et al. Intimate partner violence and women's physical and mental health in the WHO multi-country study on women's health and domestic violence: an observational study. Lancet 2008;371(9619):1165-72.

4. Dillon G, Hussain R, Loxton D, Rahman S. Mental and Physical Health and Intimate Partner Violence against Women: A Review of the Literature. Int J Family Med. 2013;2013:313909.

5. Devries KM, Mak JY, Bacchus LJ, Child JC, Falder G, Petzold M, et al. Intimate partner violence and incident depressive symptoms and suicide attempts: a systematic review of longitudinal studies. PLoS Med. 2013;10(5):e1001439.

6. Carbone-Lopez K, Kruttschnitt C, Macmillan R. Patterns of intimate partner violence and their associations with physical health, psychological distress, and substance use. Public Health Rep. 2006;121(4):382-92.

7. Mahenge B, Likindikoki S, Stockl H, Mbwambo J. Intimate partner violence during pregnancy and associated mental health symptoms among pregnant women in Tanzania: a cross-sectional study. BJOG. 2013;120(8):940-6.

8. Wathen CN, Macmillan HL. Children's exposure to intimate partner violence: Impacts and interventions. Paediatr Child Health. 2013;18(8):419-22.

9. Li Y, Marshall CM, Rees HC, Nunez A, Ezeanolue EE, Ehiri JE. Intimate partner violence and HIV infection among women: a systematic review and meta-analysis. J Int AIDS Soc. 2014;17:18845.

10. Heise L, McGrory E. Violence against women and girls and HIV: Report on a high level consultation on the evidence and its implications, 12–14 May, 2015. Greentree Estate: STRIVE Research Consortium, London School of Hygiene & Tropical Medicine; 2016 [Available from: <http://strive.lshtm.ac.uk/resources/greentree-ii-violence-against-women-and-girls-and-hiv>.

11. Ellsberg M, Arango DJ, Morton M, Gennari F, Kiplesund S, Contreras M, et al. Prevention of violence against women and girls: what does the evidence say? Lancet 2015;385(9977):1555-66.

12. Pronyk PM, Hargreaves JR, Kim JC, Morison LA, Phetla G, Watts C, et al. Effect of a structural intervention for the prevention of intimate-partner violence and HIV in rural South Africa: a cluster randomised trial. Lancet 2006;368(9551):1973-83.

13. Kim J, Ferrari G, Abramsky T, Watts C, Hargreaves J, Morison L, et al. Assessing the incremental effects of combining economic and health interventions: the IMAGE study in South Africa. Bull World Health Organ. 2009;87(11):824-32.

14. Kim JC, Watts CH, Hargreaves JR, Ndhlovu LX, Phetla G, Morison LA, et al. Understanding the impact of a microfinance-based intervention on women's empowerment and the reduction of intimate partner violence in South Africa. Am J of Public Health. 2007;97(10):1794-802.

15. Garcia-Moreno C, Jansen HA, Ellsberg M, Heise L, Watts CH, WHO Multi-country Study on Women's Health and Domestic Violence against Women Study Team. Prevalence of intimate partner violence: findings from the WHO multi-country study on women's health and domestic violence. Lancet 2006;368(9543):1260-9.

16. Ministry of Health, Community Development, Gender, Elderly and Children [Tanzania mainland], Ministry of Health [Zanzibar], National Bureau of Statistics, Office of the Government Statistician, ICF. Tanzania Demographic and Health Survey and Malaria Indicator Survey 2015-16. Dar es Salaam, Tanzania,. Rockville, Maryland, USA; 2016.

17. Harvey S, Lees S, Mshana G, Pilger D, Hansen C, Kapiga S, et al. A cluster randomized controlled trial to assess the impact on intimate partner violence of a 10-session participatory gender training curriculum delivered to women taking part in a group-based microfinance loan scheme in Tanzania (MAISHA CRT01): study protocol. BMC Women's Health. 2018;18(1):55.

18. Levack A, Rolleri L, DeAtley J. Gen.M: A Gender Transformative Teenage Pregnancy Prevention Curriculum. New York; 2014.

19. EngenderHealth. CoupleConnect: A Gender-Transformative HIV Prevention Curriculum for Tanzanian Couples May 2014 [Available from: <https://www.engenderhealth.org/files/pubs/project/champion/CHAMPION-Brief-3-CoupleConnect_lowres.pdf>.

20. Population Council. It’s All One Curriculum: Guidelines and Activities for Unified Approach to Sexuality, Gender, HIV, and Human Rights Education [Available from: <http://www.popcouncil.org/research/its-all-one-curriculum-guidelines-and-activities-for-a-unified-approach-to->.

21. Raising Voices. SASA! [Available from: <http://raisingvoices.org/sasa/>.

22. The ACQUIRE Project, EngenderHealth., Promudo. Engaging Boys and Men in Gender Transformation: The Group Education Manual New York2008 [Available from: <http://www.acquireproject.org/archive/files/7.0_engage_men_as_partners/7.2_resources/7.2.3_tools/Group_Education_Manual_final.pdf>.

23. Chan AW, Tetzlaff JM, Altman DG, Laupacis A, Gotzsche PC, Krleza-Jeric K, et al. SPIRIT 2013 statement: defining standard protocol items for clinical trials. Ann Intern Med. 2013;158(3):200-7.

24. Watts C, Heise L, M. E, Garcia-Moreno C. Putting Women First: Ethical and Safety Recommendations for Research on Domestic Violence Against Women: World Health Organisation. Geneva; 2001.

25. Ellsberg M, Heise L. Researching Violence Against Women: A Practical Guide for Researchers and Activists. Washington DC; 2005.

26. Abramsky T, Devries K, Kiss L, Nakuti J, Kyegombe N, Starmann E, et al. Findings from the SASA! Study: a cluster randomized controlled trial to assess the impact of a community mobilization intervention to prevent violence against women and reduce HIV risk in Kampala, Uganda. BMC Med. 2014;12:122.

27. Wagman JA, Gray RH, Campbell JC, Thoma M, Ndyanabo A, Ssekasanvu J, et al. Effectiveness of an integrated intimate partner violence and HIV prevention intervention in Rakai, Uganda: analysis of an intervention in an existing cluster randomised cohort. Lancet Glob Health. 2015;3(1):e23-33.
