# Supplementary material for: Pathways to reduced physical intimate partner violence among women in north-western Tanzania: Evidence from two cluster randomised trials of the MAISHA intervention
Source: PLOS Glob Public Health. 2023 Nov 13;3(11):e0002497. doi: 10.1371/journal.pgph.0002497 (PMC10642778; doi:10.1371/journal.pgph.0002497)
Supplement: Attachment — Submitted filename: Pathways_Response to reviewers_PLOSgh.docx [file pgph.0002497.s009.docx]

**Response to reviewers**

We would like to thank the reviewers for their helpful feedback. We have revised our manuscript accordingly and provide detailed responses to comments below:

**Reviewer #1:**

I congratulate the authors on a well-executed study and clearly written manuscript. The manuscript explored the pathways to reductions in physical IPV experience in two trials where the 10 session Maisha curricular was delivered i.e CRT01 involving women in existing micro-finance groups that were doing well with their loan repayments and CRT02 involving women recruited from the community and with no prior micro-finance group exposure. Positive impacts of intervention were reported in CRT01 and not CRT02. Sub-analyses showed that impacts in CRT01 were greater/significant among younger women who had no IPV exposure at baseline and no impacts recorded among women with IPV exposure at baseline or violent relationships. Mediator analyses implicated attitudinal changes i.e reduced acceptability of IPV as critical for observed reduced risk of physical IPV i. CRT01. Other anticipated mediator pathways were not established e.g improved communication was not associated with positive intervention impacts but those that had better communication at baseline had reduced physical IPV risk. Overall, the authors highlight the importance of structural intervention and women being less financially dependent in preventing the onset of IPV risk overall especially among younger women through low acceptability of IPV, decisions they make about partners and better communication that mitigates exacerbation of arguments to conflict and IPV. They also clearly identify the limitations of the study including the measurement of mediators cross-sectionally and the impossibility of establishing the directionality of some of the assumed associations/ pathways. I have few comments for the authors to consider attending to:

Thank you for this nice summary of the paper and your encouraging comments. We also appreciate the constructive criticism that you have offered, to which we provide detailed responses below.

Background/Introduction
1. The manuscript is written in a style that assumes that the audience are familiar with the context of the study sites in Tanzania. However, my view is that it may be worthwhile to provide some context specific description to better understand some of the conceptualisation of pathways to change and intervention. For example, how does the context’s social organisation and culture relate to the fluidity of relationships or the stability of marriages or living together as married or the dynamics relevant to dissolving relationships.

Thank you for pointing out this omission. We have now added a paragraph on context to the start of the Material and Methods section, under the level-2 heading ‘Study setting and study design’.

Methods
2. For the mediator analysis, can the authors specify and reference how they used cutoffs to determine whether mediator effects were small, weak or large in the data analysis section.

Thank you for this question. We have not categorically defined mediator effects as small, weak or large. Rather, as specified in the statistical analysis section, we interpret “greater attenuation as suggestive of the increased importance of that variable as a mediator of intervention impact on IPV”. We are concerned that any cut-offs would be arbitrary (in the same way that we do not tend to rigidly define odds ratios themselves as large or small). Levels of attenuation will reflect the number of people for whom the mediator has played a role (not all pathways will be relevant to all people), in addition to the size of the role each mediator has played for those people.

3. Can the authors provide more detail about the criteria for selection of neighbourhood in CRT02 rather than just saying “suitable neigbourhoods”

We have now provided additional information in two places in the manuscript. First, in the section describing the overall design of each trial, we have specified: “The CRT02 trial was also conducted in Mwanza city but in different wards where BRAC (the microfinance provider in CRT01) was known to be less active.“ We have also added text to the paragraph headed “CRT02 – neighbourhood groups“, in which we describe efforts to identify neighbourhoods that are similar to those included in CRT01, with respect to levels of urbanisation.

Results
4. Line 304- Can the abbreviation LRT be written in full first time.

Likelihood ratio tests are first referred to in full in line 268 (tracked version). However, we had forgotten to include the acronym in brackets after this first use of the term. Thanks for pointing out this omission. We have now corrected this.

5. Table 1 presents disaggregated %s of different participant characteristics in CRT01 & CRT02. However, it does present data to indicate that tests for statistical significance of differences in proportions were conducted. Given the different sample sizes in the 2 trials, such tests can conclusively support whether the samples were statistically different. The text accompanying the table implies significant difference, but the results of statistical tests are needed as evidence of this.

Thank you for this comment. We had not presented tests of statistical significance of differences between the two trial populations as the comparison was made in order to identify differences that might be ‘clinically’ important (in terms of potential to influence observed intervention effectiveness) between our two samples. We would argue that it is the magnitude of the *observed* differences in our samples that is important from this perspective, regardless of whether these differences arose by chance (sampling error) or reflected ‘real’ (statistically significant) differences between the two populations from which our trial participants were drawn. If you are not satisfied with this reasoning, we are happy to reconsider their inclusion. However, it was a deliberate decision not to include tests of statistical significance rather than an oversight.

Discussion
6. Given the reported impacts of intervention, it is important that the authors consider adding text that emphasises the limitation/ challenges of not working with male perpetrators of IPV to address IPV continuation among abused women. Currently the manuscript has emphasised that the VAW field prioritises economic empowerment programmes targeting women to reduce IPV. Yet the data presented also shows some unintended impacts among CRT01 women such as the backlash of increased arguments over gender roles and other conflicts. Ultimately CRT02 worked with women addressing their individual vulnerabilities to IPV but this was insufficient to lead to positive relational and IPV impacts without the economic empowerment and the non-involvement of male partners.

I have addressed points 6 and 7 in my response below point 7.

7. The authors refer to similar barriers observed with other interventions such as IMAGE and SASA. In my view it is warranted that the authors reflect and provide some answers/suggestions to questions such as “How should the field pivot in terms of interventions that address ongoing IPV and among older women?” What design or content issues require emphasis/ attention or inclusion” “How could IPV interventions with economic empowerment components have maximised impacts and brought to scale in LMIC settings?”

You raise a really important issue. Below we highlight where we have addressed this issue in the Discussion, and additions we have made to the manuscript in response to your comment:

- In relation to participation in community meetings being associated with increased risk of IPV, we state “This suggests that interventions with men and others in the community are necessary in order to increase partner- and community-level acceptance of women taking on more active roles outside of the house and within the community.“
- We state that changes to women’s attitudes and behaviours, in the absence of any intervention with male partners, may increase tension in less equitable relationships, and go on to say: “The importance of involving men in IPV prevention programming, including through couples-based interventions, has gained traction in recent years, and CRTs of several such interventions have demonstrated their effectiveness in preventing IPV.“ We provide references for several such CRTs, and have added in the specific example of Indashyikirwa, a couples based intervention in Rwanda that led to substantial reductions in IPV among recipients.
- In relation to the possible increase in women reporting that their partners often suspect them of infidelity, we again discuss the potential to avoid backlash and increase intervention impacts “if men’s attitudes linking infidelity to the acceptability of women refusing sex or seeking work outside the home were addressed.” We have now added to our description of the SASA! intervention and trial, as this is a counter example which did involve men and women and did manage to reduce levels of suspicion over infidelity: “Indeed, in the SASA! trial of a community intervention that involved entire communities – both women and men – to address community-level norms underpinning high levels of VAW, levels of male partners’ suspicion over infidelity did fall. This fall came alongside progressive shifts in both women’s and men’s attitudes around IPV and women’s right to refuse sex. The intervention led to community-level reductions in IPV, with changes in men’s attitudes and reduced suspicion over infidelity emerging as potentially important mediators of this impact.“
- The two final paragraphs of our Conclusions are focused entirely on the need for IPV prevention interventions to address structural and socio-ecological (including partner and relationship-level) factors in addition to individual-woman-level factors. The penultimate paragraph addresses opportunities for incorporating economic strengthening programming into IPV prevention programming. We have expanded on this to discuss types of economic interventions that have been successfully combined with social empowerment components in the prevention of IPV, as well as opportunities these may provide for scaling up IPV prevention programming.
- Our final paragraph addresses issues of limited observed relationship-level change, issues of backlash, and the need for IPV prevention to include men and others in the community. We have now expanded this paragraph to cite an example of how the involvement of men and more diverse community actors may be particularly important for the prevention of ongoing abuse.

**Reviewer #2:**

The main comments are about the randomization. Other comments include sample construction and considering the endogeneity of the results.

Thank you for your feedback and helpful comments. We have provided a detailed response to each point below.

This paper studies the effectiveness of interventions that target women's empowerment

through standalone social empowerment interventions compared to those that also target

their financial vulnerability, especially their dependence on male members of the household.

The study attempts to parse out the importance of interventions that alleviate women's

financial dependency by specifically looking at these interventions. The outcome of interest

is physical Intimate Partner Violence (IPV). The authors find that, on average, the inter-

ventions that include an economic empowerment component, i.e., access to microfinance

in this case, reduce physical IPV a lot more than interventions without. These effects are

concentrated among those who are young, more financially independent, and have no prior

exposure to physical IPV. The results from the study highlight the importance of including

economic and financial empowerment components for women to reduce the acceptance of IPV and hence reduce the incidences of IPV.

Thank you for this summary, which nicely captures the implications of our findings. In relation to this summary and further comments below, I would like to clarify that neither trial was set up to evaluate the effectiveness of financial empowerment for the prevention of IPV. Both trials evaluated the same ***gender training intervention***. The difference between them is that they assessed the impact of the gender training among different populations of women, CRT01 among women receiving microfinance (both control and intervention arm received microfinance) and CRT02 among women not receiving microfinance (neither the control nor intervention arm received microfinance). The question we can attempt to answer based on the two trial results is, therefore, whether the impacts of gender training on IPV prevention are somehow facilitated by women’s financial empowerment. We cannot answer based on these trials whether microfinance itself is an effective prevention strategy.

Major Comments

1. My main concern is about the treatment and control groups, especially in CRT01. On

page 5, in the Section on Materials and Methods, the authors describe the groups in

the following manner:

• CRT01: Out of the 66 existing microfinance group: 33 groups received the gender

intervention, whereas 33 didn't (they were waitlisted for the future)

• CRT01: Out of the 66 groups that were not involved in microfinance loans: 33

groups received the gender training intervention, and 33 were waitlisted (control).

In the analyses, when the authors mention the effects of the interventions for the CRT01

trial - do they refer to the control group as the CRT01 group that was waitlisted, or is

the control group in the CRT02? The interpretations need to be a lot more explicit,

since it is unclear what the correct control group is from the explanation. The figures

explain this better.

Thank you for this comment. We had also referred to the waitlisted groups in CRT01 as ‘control’ groups in the methods section. However, in case this was not clear we have now amended the methods text to read:

“The CRT01 trial recruited 66 existing microfinance loan groups. Of these, 33 groups (544 women) were randomised to receive the MAISHA gender training intervention, and 33 groups (505 women) were waitlisted to receive the intervention after trial completion (control). In the CRT02 trial, the trial team worked in collaboration with local leaders to form 66 neighbourhood groups of women not engaged in a microfinance loan scheme. Of these, 33 groups (627 women) were randomised to receive the MAISHA gender training intervention, and 33 groups (638 women) were waitlisted (control).“

Each trial is a standalone trial so they each have their own intervention arm and their own control arm. We do not perform any analysis comparing the intervention arm in one trial to the control arm in the other.

2. Relatedly, if it is the case that the 33 existing microfinance groups that did not receive

the gender intervention are in fact the control groups, then I am a bit unsure about

about the results of the intervention. Given that this set of women were aware that

they were going to receive the intervention after the trial ended, the anticipation effect

of the program could contaminate the results. The spillover from the expectation is in

fact something to consider in these results.

Thank you for this comment. As you point out, anticipation of participation could potentially affect attitudes and behaviours. This is the same in both of our trials (the control arm were waitlisted in both CRT01 and CRT02) so doesn’t affect our comparison of intervention impacts between the two trials.

Waitlisting the control arm in a trial of a social intervention is fairly common practice, and indeed often a recommendation of IRBs so as not to deprive trial participants of potentially beneficial interventions in the longer term. In stepped wedge trials, in fact, the staggering of intervention delivery to all participants in the trial is an integral part of the trial design. Arguably, since we want to test the effectiveness of the actual intervention (rather than the idea of receiving an intervention), it adds to internal study validity if the control arm also has some sense of participating in an intervention (while not actually receiving the specific intervention under study) – in the same way that control participants in clinical trials receive a ‘placebo’ treatment.

3. The CRT01 results do not seem surprising because these group of women were already

exposed to financial empowerment through access to microfinance loans. A cleaner

control group would have been a group of women who had no established microfinance

association, no gender intervention, and no anticipation of any of these interventions.

This would give us a true understanding of the mechanisms.

Both trials (CRT01 and CRT02) were set up to evaluate the ***impact of a gender training intervention on prevention of IPV***. Neither trial was set up to evaluate the impact of microfinance on IPV prevention (in CRT01 both the intervention and control arm were receiving microfinance, and in CRT02 neither the intervention nor control arm were receiving microfinance). The difference between CRT01 and CRT02 is that they evaluate ***the effectiveness of gender training*** among two different populations of women, the first among women receiving microfinance, and the second among a population of women not receiving microfinance. Thus, the question we can answer by comparing results between the two trials (not just by considering CRT01 in isolation) is whether microfinance appears to facilitate ***the impact of a gender training intervention on prevention of IPV***. At no point do we try to answer whether microfinance itself is an effective prevention strategy.

4. Additionally, it would have been more beneficial to see if groups that had the gender

intervention, received a microfinance intervention rather than the other way around.

This would give us the true effect of an economic empowerment intervention.

Thank you for this comment. As my above response indicates, our intention was not to evaluate the effect of an economic empowerment intervention. The effectiveness of economic empowerment interventions is an interesting question which has been the subject of other research we and others have been involved in (as referenced in the background section). In this piece of research, however, we examine mechanisms of effectiveness of a gender training intervention, also an important area of research since group-based gender training is a widely adopted intervention model.

5. It is important to see whether the pre-treatment baseline characteristics are similar

between the treatment and the control groups. This will inform the readers that the

randomization was done correctly. Currently, in Table 2, only the baseline character-

istics are mentioned. It would be informative if the authors provided t-statistics of the

differences between the treatment and control groups to show that the randomization

was done correctly. Of course, it maybe the case that the key outcome variable could

be significantly different between the control and treatment group.

This is an important point, which we thank you for raising. In our first results paragraph we have the sentence: “In both trials, intervention and control arm women were similar with respect to a range of baseline characteristics“, and we reference the original trial papers in which the disaggregated intervention and control arm baseline data are presented. We have now included these tables as a supplementary table (S4 Table) in this paper so as to reassure the reader of the internal validity of the two trials that are being summarised and compared in this paper. We have also added which variables demonstrated some imbalance. We would like to highlight that, as detailed in our methods section, our analysis of intervention effect is adjusted for age, education and baseline measure of the outcome.

3 Other Comments

1. In the sample, there seem to be unmarried women, and unmarried women who are living

with their partners, unless I am misunderstanding this. For the unmarried women not

living as if married, who are they facing the physical IPV from? This portion was not

clear.

Thank you for this question. The IPV questions are asked of all women who have ever had a partner (including non-cohabiting partners). We have now added this explanation into the Outcome sub-section of the Materials and Methods section. The IPV outcome is not confined to married/cohabiting women as women may be in intimate relationships in which they are not married/cohabiting but are still subjected to violence by their partner. It is also important to measure violence among women who have been in relationships in the past even if they are not currently in a relationship, as women may be at high risk of (continued) violence from past partners. As specified in Table 1, the violence questions are worded: “Has your current partner or any other partner…”

2. One of the reasons that women may be more likely to be accepting IPV is if they have

children. While the tables on potential mediators report on \Argued with partner

over him treating her/ her children disrespectfully", the analyses does not specifically

report on whether women's attitudes differ based on whether they have kids or not,

and whether they have multiple kids.

This is an interesting idea. While it is not our intention in this paper to explore factors associated with different attitudes on IPV, it is true that whether or not women have children can affect their attitudes/acceptance of IPV as well their capacity to leave a violent relationship. Therefore, this would be a potential effect modifier of intervention effectiveness. We have not included it in our analysis of effect modification due to extremely low numbers of women with no children, but we have now added data on children to our baseline table (Table 2) and results text, and noted the omission of children in our analysis of effect modification as a limitation (in the discussion).

3. Were some women unmarried prior to the treatment and then got married during the

study? This could have potential implications on the results seen in the study, espe-

cially for those in CRT01. The choice of spouse can depend on women being financially

independent. Women who are financially independent have higher autonomy and are

more likely to choose spouses who are similar to them in beliefs. Since these women

already have access to microfinance loans, they are less likely to be dependent on their

future spouses, and choose spouses who are more aligned with their beliefs and who

don't engage in IPV (this could be unknown, of course).The study's results need to be evaluated in this context.

Thank you for raising this issue. We had initially hoped to explore the role of women’s partner choice as a mechanism of intervention effect (both women’s propensity to enter into new relationships, and changes in women’s criteria for choosing new partners). As you say, women’s capacity to change their behaviour and choices regarding new partners in response to the intervention is something that might be modified by their financial autonomy. However, we were not able to look at this factor as very few women entered into new relationships during the course of the study. We have now noted our inability to explore this factor as a limitation (in our discussion section).
